# Global misregulation of genes largely uncoupled to DNA methylome epimutations characterizes a congenital overgrowth syndrome

**DOI:** 10.1038/s41598-017-13012-z

**Published:** 2017-10-04

**Authors:** Zhiyuan Chen, Darren E. Hagen, Tieming Ji, Christine G. Elsik, Rocío M. Rivera

**Affiliations:** 1 Division of Animal Sciences, University of Missouri, Columbia, MO 65211 USA; 2 Department of Statistics, University of Missouri, Columbia, MO 65211 USA; 3 ZC–159 G Warren Alpert Building, 200 Longwood Avenue, Boston, MA 02115 USA; 4 Present Address: Department of Animal Science, Oklahoma State University, 311C Noble Research Center, Stillwater, OK 74078 USA

## Abstract

Assisted reproductive therapies (ART) have become increasingly common worldwide and numerous retrospective studies have indicated that ART-conceived children are more likely to develop the overgrowth syndrome Beckwith-Wiedemann (BWS). In bovine, the use of ART can induce a similar overgrowth condition, which is referred to as large offspring syndrome (LOS). Both BWS and LOS involve misregulation of imprinted genes. However, it remains unknown whether molecular alterations at non-imprinted loci contribute to these syndromes. Here we examined the transcriptome of skeletal muscle, liver, kidney, and brain of control and LOS bovine fetuses and found that different tissues within LOS fetuses have perturbations of distinct gene pathways. Notably, in skeletal muscle, multiple pathways involved in myoblast proliferation and fusion into myotubes are misregulated in LOS fetuses. Further, characterization of the DNA methylome of skeletal muscle demonstrates numerous local methylation differences between LOS and controls; however, only a small percent of differentially expressed genes (DEGs), including the imprinted gene *IGF2R*, could be associated with the neighboring differentially methylated regions. In summary, we not only show that misregulation of non-imprinted genes and loss-of-imprinting characterize the ART-induced overgrowth syndrome but also demonstrate that most of the DEGs is not directly associated with DNA methylome epimutations.

## Introduction

The use of ART has become increasingly common worldwide and each year approximately six percent of infants born in developed countries are conceived employing these technologies^1^. The use of ART involves manipulation of gametes and/or embryos in an artificial environment, and it has been reported that ART-conceived children have increased risk for birth defects^2^. For example, a meta-analysis of eight epidemiologic studies indicates that ART-conceived children have a 5.2-fold increased likelihood of developing the congenital overgrowth condition BWS^3^. However, it is still unresolved whether the use of ART or the infertility per se is the main cause of the loss-of-imprinting disorders observed in ART-conceived humans^3^. BWS is characterized by complex and variable symptoms including pre- and post-natal overgrowth, enlarged tongue, ear malformation, umbilical hernia, and predisposition to develop childhood tumors^4^. In ruminants, gametes and embryos subjected to *in vitro* manipulations can develop into unusually large offspring that share phenotypes with BWS^5,6^. LOS, as the congenital overgrowth syndrome is referred to in ruminants, can cause detrimental effects to both the dam and offspring such as delivery difficulty due to the oversized fetus and inability for the newborn to stand and to suckle^6^. It is unclear what triggers the development of these congenital overgrowth conditions and their associated phenotypes and why ART potentiates the syndromes. Serum supplementation of the oocyte maturation medium and the embryo culture medium has been recognized as a mediator of LOS^6^, although the factors in the serum that are responsible for the overgrowth phenotype remain to be identified. Several animal studies have indicated that the use of ART can alter the epigenome of the gametes and embryos^7–10^ and this can contribute to the etiology of the ART-induced overgrowth conditions^3,11^.

DNA methylation is an epigenetic modification involving the addition of a methyl group to the 5^th^ carbon of cytosine^12^. In mammals, DNA methylation typically occurs in a CpG context with the exception of CpH methylation, which is mostly observed in neural tissues, oocytes and embryonic stem cells^13,14^. DNA methylation plays a key role in many biological processes such as regulation of tissue-specific gene expression, suppression of parasite DNA in the genome, X-chromosome inactivation, and genomic imprinting^12^. Genomic imprinting is an epigenetic phenomenon in which a subset of genes, known as imprinted genes, are transcribed monoallelically in a parental-origin-dependent manner^15^. The transcriptional asymmetry of the parental alleles is usually directed by allele-specific DNA methylation (ASM) at imprinted loci established during gametogenesis^15^. Proper allele-specific DNA methylation and allele-specific expression of imprinted genes play important roles in the regulation of embryonic and neonatal growth, placental function, postnatal behavior, and metabolism^16^. Altered gene expression and DNA methylation at imprinted loci have been associated with congenital overgrowth disorders such as BWS in human^17^ and LOS in bovine^5,18^.

Most BWS cases are sporadic and have been linked to two imprinted loci on chromosome 11p15.5, the *KCNQ1* locus and the *H19/IGF2* locus^4^. Approximately 50% of the BWS cases are associated with the hypomethylation of KvDMR1 at the *KCNQ1* locus and 2–7% are linked to the hypermethylation of the differentially methylated region (DMR) at the *H19/IGF2* locus^4^. In addition, studies have shown that a subset of BWS patients with epimutation at the *KCNQ1* locus also exhibit abnormal DNA methylation at other imprinted loci^19^. We have observed loss of methylation of the KvDMR1 on the maternal allele in LOS^5^ and have reported that LOS is a multi-locus loss-of-imprinting condition in which aberrant imprinted gene expression is associated with tissue-specific loss of imprinted DNA methylation^18^.

Although it is well accepted that loss-of-imprinting can contribute to these overgrowth syndromes, and as such have been coined “loss-of-imprinting syndromes”, it remains unknown whether aberrant gene expression and DNA methylation occur at non-imprinted loci and to what extent these molecular alterations contribute to the variable phenotypes observed in these conditions. To address this question, we examined the transcriptome of skeletal muscle, liver, kidney, and brain of four control and four LOS day ~105 *Bos taurus indicus* (*B*. *t*. *indicus*) × *Bos taurus taurus* (*B*. *t*. *taurus*) F_1_ fetuses^18^. We found that different LOS fetuses exhibit different numbers of DEGs and that each tissue within LOS fetuses have perturbations of distinct gene pathways. Notably, in skeletal muscle, multiple pathways involved in myoblast proliferation and fusion into myotubes are misregulated in LOS fetuses. Further, characterization of the DNA methylome of skeletal muscle revealed numerous local methylation differences between LOS and controls; however, very few DEGs could be linked to neighboring identified DMRs. This study indicates that global misregulation of non-imprinted genes in addition to loss-of-imprinting characterizes the ART-induced overgrowth syndrome. The observation that most identified DMRs could not be directly associated with aberrant gene expression suggests that caution should be exercised when making conclusions about the etiology of such syndromes by interpreting time-point-specific DNA methylation data.

## Results

### Identification and characterization of DEGs in LOS fetuses

In order to determine to what extent the transcriptome is altered in bovine LOS fetuses, we analyzed RNA sequencing (RNAseq) data of skeletal muscle, liver, kidney, and brain from four control and four LOS day ~105 (d104–106) *B*. *t*. *indicus* × *B*. *t*. *taurus* F_1_ fetuses that we generated in a previous study^18^ (Supplementary Table 1). These tissues were selected because they represent the three primary germ layers (*i*.*e*., liver for endoderm, kidney and skeletal muscle for mesoderm, and brain for ectoderm). Only females were used in this study to avoid any potential sex-specific gene expression^20^. The average bodyweight for control and LOS fetuses was 405 g [standard deviation (SD) = 10 g, range = 24 g] and 592 g (SD = 95 g, range = 200 g), respectively (p = 0.008) (Supplementary Table 1). The bodyweight differences between the control and LOS fetuses are not expected to be due to paternal effects because all fetuses were sired by one Nelore bull [ABS CSS MR N OB 425/1 677344 29NE0001 97155 (*i*.*e*., *B*. *t*. *indicus*)]^5^.

As LOS fetuses exhibited dramatic difference in bodyweight, each LOS fetus was considered individually and was compared to the mean of the four controls to identify DEGs. To ensure the validity of grouping four control fetuses for DEG identification, principle component analyses (PCA) were performed using the normalized RNAseq read counts. For the RNAseq﻿ th﻿e﻿ expression libraries of kidney, skeletal muscle, and liver, control fetuses clustered while the libraries from the LOS fetuses did not (Supplementary Fig. S1). It should be noted that the RNAseq libraries of skeletal muscle and liver from Control #2 fetus were sequenced with 50 bp read length; while the RNAseq reads of other fetuses were of 100 bp. The read length differences may explain why Control #2 is segregated from other control fetuses in these two tissue types. In brain, both control and LOS fetuses exhibited a segregated pattern in the PCA plot (Supplementary Fig. S1). In order to minimize the false positive DEGs caused by natural biological variation and technical variation, the DEGs were identified with the consideration of variance among the controls and the RNAseq read length differences using the edgeR package as we previously described^18,21,22^. The number of DEGs varied between tissues for different LOS fetuses (Supplementary Fig. S2). For example, in the largest LOS fetus analyzed (weight = 714 g), the number of DEGs was 3868, 1581, 441, and 276 for skeletal muscle, liver, kidney, and brain, respectivley, while in the smallest LOS fetus (weight = 514 g), the number of DEGs for those tissues were 2270, 3232, 139, and 1106 (Supplementary Fig. S2). The number of DEGs was not associated with fetal weight, but the fetuses with loss-of-imprinting at the *KCNQ1* locus (*i*.*e*., LOS #1 and #4) had more DEGs in liver and skeletal muscle than LOS fetuses with correct imprinting at this locus (Supplementary Fig. S2).

**Figure 1:**
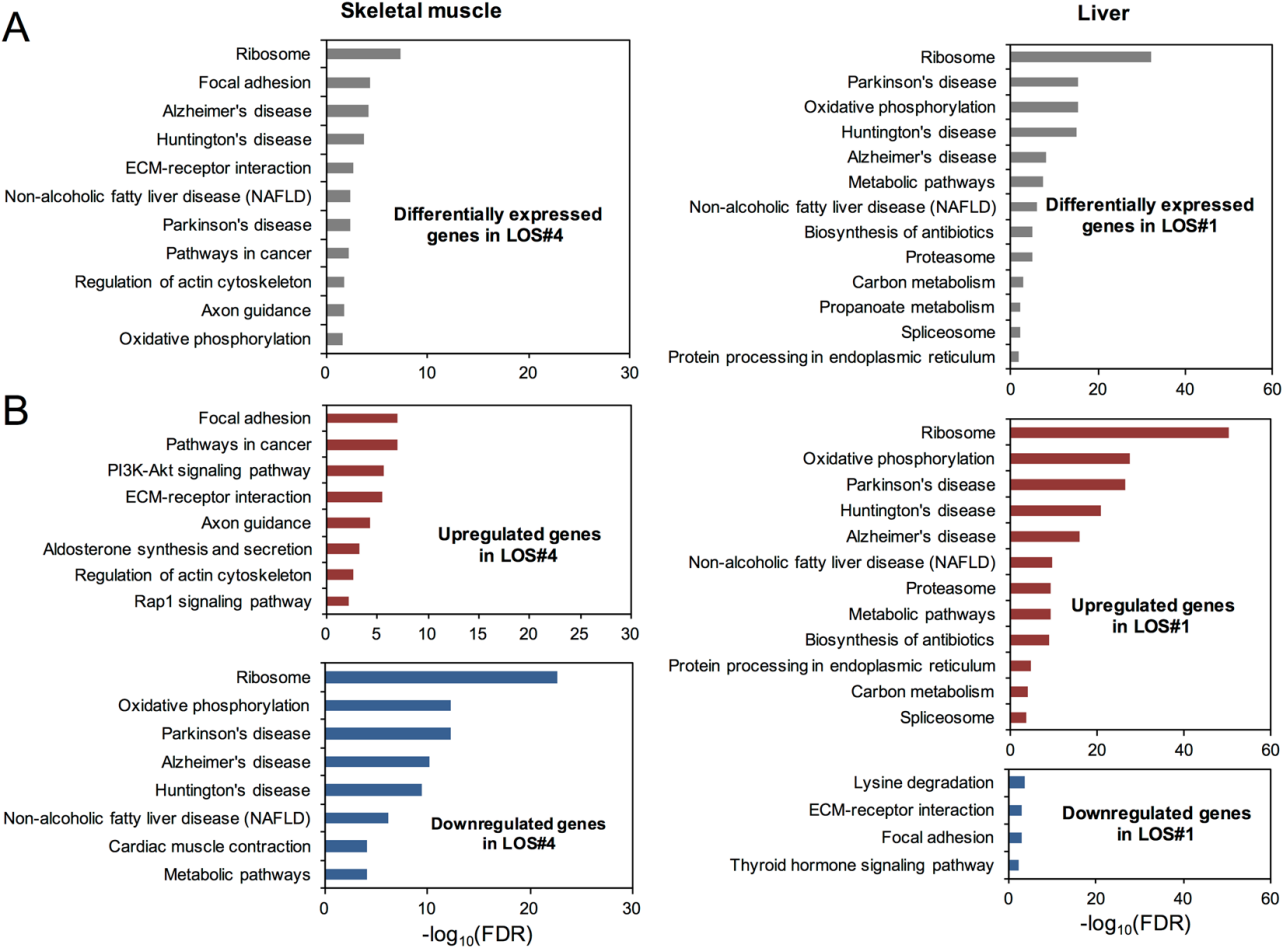
KEGG pathway analyses of DEGs in somatic tissues of LOS fetuses. (**A**) KEGG pathways enriched for DEGs in skeletal muscle and liver (FDR < 0.05). Shown here are examples of tissues with the most DEGs. (**B**) KEGG pathways enriched for upregulated and downregulated genes in skeletal muscle and liver (FDR < 0.05). DEG: differentially expressed genes; LOS: large offspring syndrome.

Next, we performed Kyoto Encyclopedia of Genes and Genomes (KEGG) pathway analyses^23^ to gain biological insights of the DEGs identified in the LOS fetuses. We found that several KEGG pathways were shared by DEGs in different tissues while others were unique for a particular tissue (Fig. 1A and Supplementary Fig. S2). For example, “ribosome” and “oxidative phosphorylation” were enriched in DEGs in skeletal muscle, liver, and kidney, while “pathways in cancer” was unique for skeletal muscle (Fig. 1A and Supplementary Fig. S2), suggesting the over-proliferation of the muscle cells in LOS fetuses. Further analyses revealed that even the common pathways could be differentially represented in different tissues. For instance, “ribosome” and “oxidative phosphorylation” were enriched with downregulated genes in skeletal muscle but enriched with upregulated genes in liver and kidney (Fig. 1B and Supplementary Fig. S2). For brain, however, misregulated genes were only found to enrich two KEGG pathways (Supplementary Fig. S2). Overall, we found that numbers of DEGs varied among LOS fetuses and different tissues within LOS fetuses have perturbations of distinct gene pathways.

### Multiple pathways involved in myoblast proliferation and fusion into myotubes are misregulated in LOS

**Figure 2:**
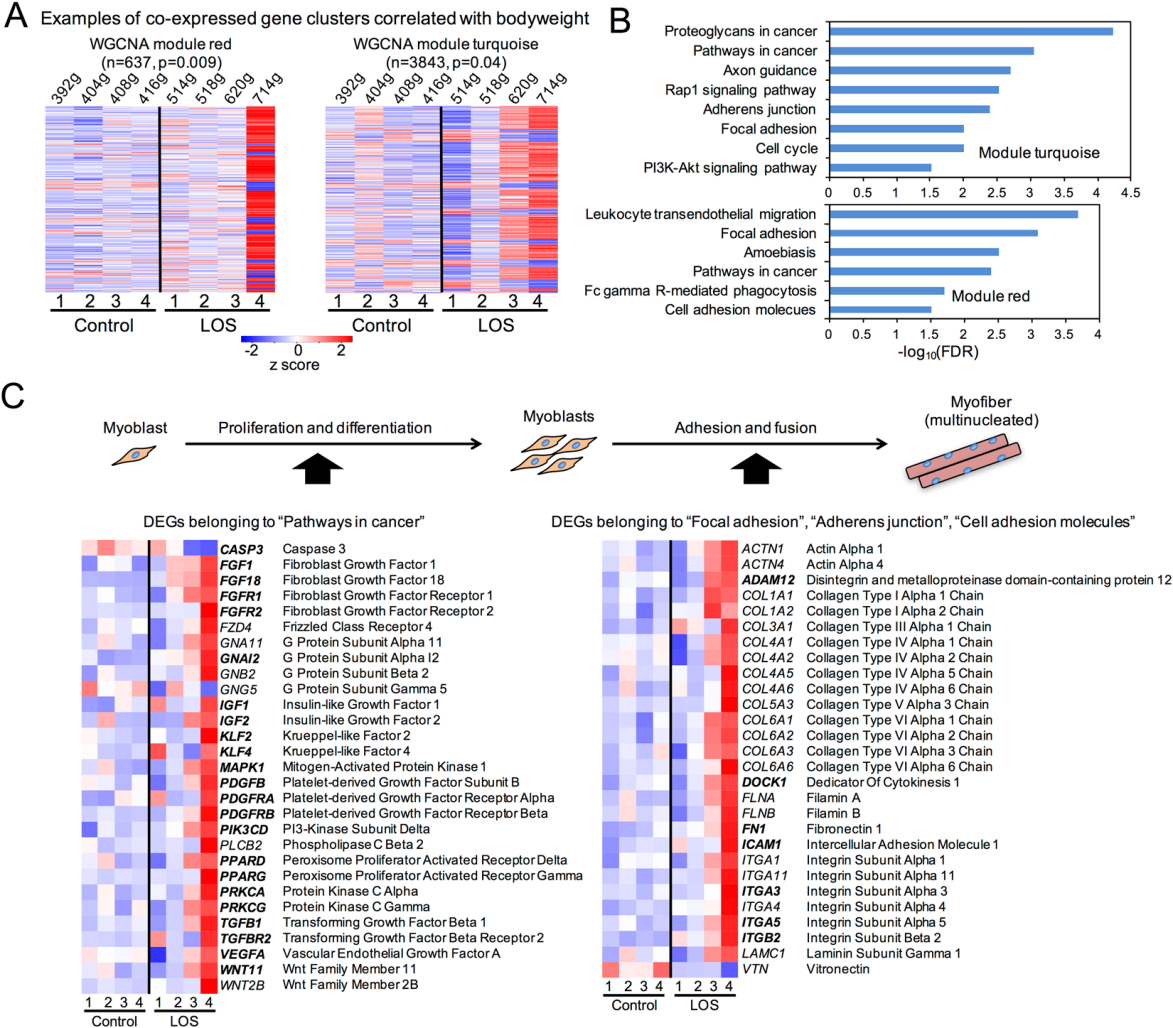
Multiple pathways involved in myogenesis are misregulated in skeletal muscle of LOS fetuses. (**A**) Heat map profiling of the genes of the WGCNA network module red and turquoise. Red and turquoise modules represent clusters of co-expressed genes that are correlated with bodyweight of the fetuses (details may be found in Fig. S2). Shown in the parentheses are the number of genes in each module and p-value of the correlation between the module and bodyweight. The z-score scale represents the mean-subtracted regularized log-transformed read counts. (**B**) Enriched KEGG pathways for module turquoise and red (FDR < 0.05). (**C**) Heat map profiling of the genes that belong to the “pathways in cancer” and the “focal adhesion/adherens junction/cell adhesion molecules” pathways. Bolded genes are those known to be involved in myoblast proliferation and differentiation or fusion of myoblasts into myotubes.

To identify DEGs correlated with the overgrowth phenotype of the LOS fetuses, weighted gene correlation network analyses (WGCNA)^24^ were used to identify clusters of genes that were highly interconnected, namely modules, and to determine which modules were correlated with bodyweight of the fetuses. For all the tissues analyzed, three modules [*i*.*e*., turquoise, red, and green (Supplementary Fig. S3)] in skeletal muscle were identified to be significantly correlated (p-value < 0.05) with bodyweight and were enriched with the KEGG pathways associated with overgrowth phenotype (Fig. 2A,B). In both turquoise and red clusters, the enriched pathways were involved in cell proliferation and differentiation (*e*.*g*., “pathways in cancer” and “cell cycle”), and cell-cell adhesion and fusion (*e*.*g*., “focal adhesion”, “cell adhesion molecules”, and “adherens junction”) (Fig. 2A,B), while the green module was only enriched with genes associated with the “spliceosome” pathway (Supplementary Fig. S3).

Fetal myogenesis involves myoblast proliferation/differentiation and fusion of myoblasts into multinucleated muscle fibers^25^. Characterization of genes that belong to “pathways in cancer” led us to identify multiple upregulated genes in LOS fetuses that are known to be involved in myoblast proliferation and differentiation (Fig. 2C). For example, IGF1 is a major regulator for skeletal muscle growth and both *IGF1* ^26^ and its downstream signaling component *PIK3CD* ^26^ exhibited increased expression levels in LOS #3 and/or #4, the two largest fetuses. Besides *IGF1*, transcript abundance of several other growth factors and their receptors such as *FGF1*, *FGF18*, *FGFR1*, *FGFR2*, *PDGFB*, *PDGFRA*, *PDGFRB*, *TGFB1*, and *TGFBR2* were also increased in LOS #3 and/or #4 (Fig. 2C). These growth factors may work in synergy to promote myoblast proliferation and differentiation^25^. In addition, numerous upregulated genes in LOS that belong to the “focal adhesion” and the “cell adhesion molecules” pathways have been reported to play a role in myoblast adhesion and fusion. For example, we found *DOCK1*, a prototypical member of the family of Rho GTPase activator that is essential for myoblast fusion^27^, to be upregulated in both LOS #3 and #4 (Fig. 2C). Further, a number of extra cellular matrix genes, reported to be involved in myoblast migration, adhesion, and fusion (*e*.*g*., *ADAM12*, *FN1*, *ICAM1*, *ITGA3*, and *ITGA5*)^28,29^ exhibited increased transcript abundance in LOS #3 and/or #4 (Fig. 2C). Although it is likely that some of the gene expression changes could be the consequential events of the altered fetal myogenesis, these data suggest that the misregulated pathways may be responsible for the increased muscle mass and overgrowth phenotype of the LOS fetuses. In summary, gene co-expression network analyses revealed that multiple pathways involved in fetal skeletal muscle development were disrupted in LOS.

### Whole genome bisulfite sequencing (WGBS) and read processing

As differential gene expression can be regulated by differential DNA methylation^12^ and DNA methylation can be altered by the use of ART^7–10^, we next sought to address whether altered DNA methylation could be the molecular mechanism responsible for the misregulated gene pathways in skeletal muscle of LOS fetuses. To compare the methylome between control and LOS, we generated WGBS data of skeletal muscle of the fetuses used for RNAseq analyses. For each individual, we sequenced ~400 million 100 bp paired-end reads, which is approximately 26 × coverage of the bovine reference genome (Supplementary Table S1). The average bisulfite conversion rate was 99.31% (Supplementary Table S1). Of note, strong methylation bias was detected at the 5′ end of read mate 1 and both ends of read mate 2 (Supplementary Fig. S4). As these biases could be introduced during the library preparation and/or sequencing steps^30^, the first 3 base pairs (bp) and last 2 bp of each WGBS read were trimmed and not used for determination of CpG methylation levels. Following a series of quality filtering steps, ~80% of the CpGs in the reference genome were covered by ≥ 5 WGBS reads (Supplementary Table S2).

### Genome-wide CpG methylation landscape and its relationship with transcriptome in fetal skeletal muscle

**Figure 3:**
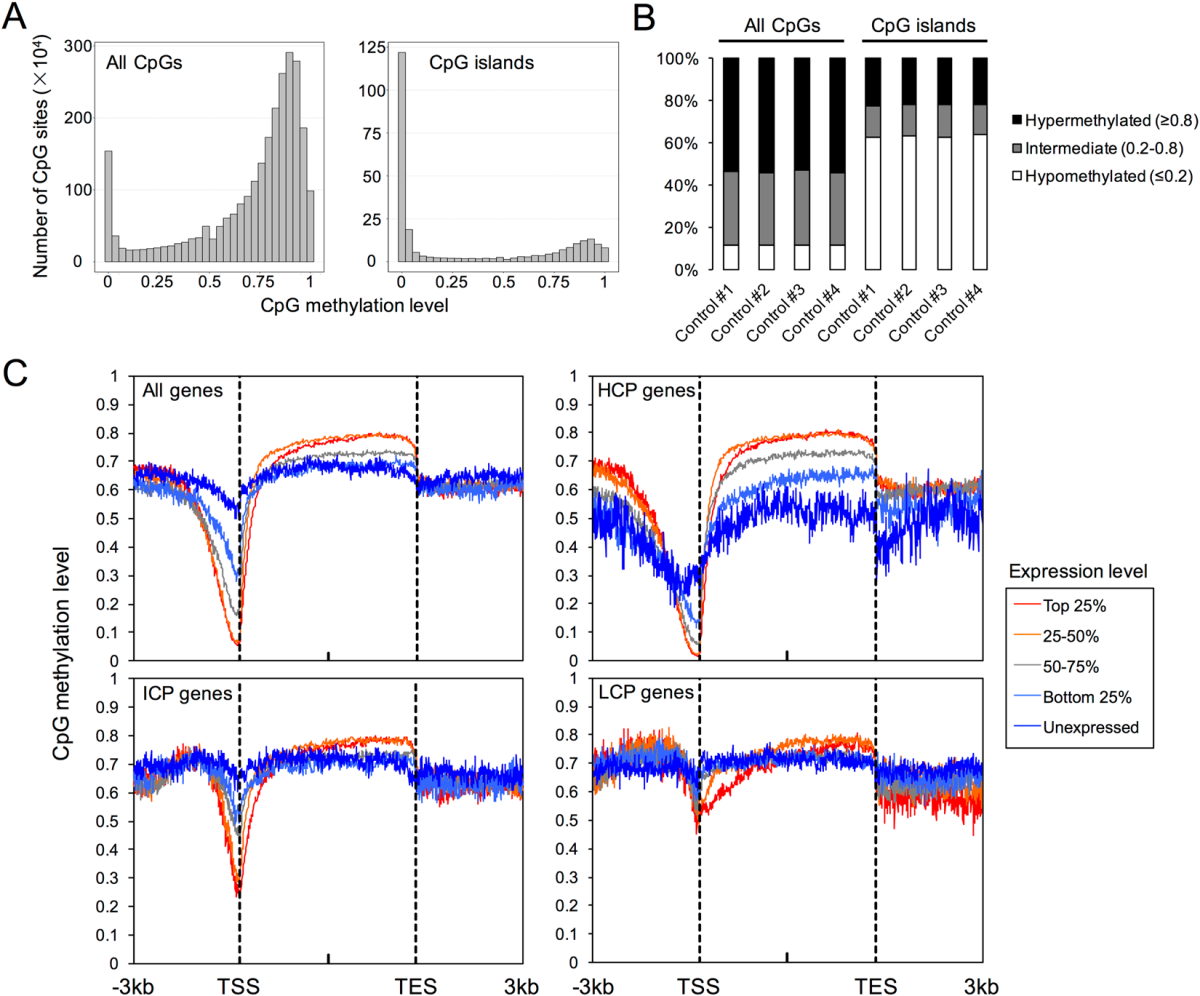
Genome-wide CpG methylation landscape and its relationship to the transcriptome in day ~105 skeletal muscle of control fetuses. (**A**) Distribution of CpGs sites across different methylation levels. The methylation level of each CpG site was calculated as the ratio of the “C” reads to the sum of the “C” and “T” reads. CpGs from all four controls were combined for the generation of this plot. (**B**) Percent of CpGs at each methylation level in control fetuses. (**C**) Averaged DNA methylation of annotated bovine genes along the gene body and 3 kb upstream of the TSS and 3 kb downstream of the TES. Genes were classified into five groups based on their expression levels. HCP: high CpG density promoter; ICP: intermediate CpG density promoter; LCP: low CpG density promoter; TSS: transcription start site; TES: transcription end site.

Prior to making comparisons between LOS and controls, we sought to establish the DNA methylation baseline in bovine fetal skeletal muscle. In controls, the distribution of CpG sites across different methylation levels exhibited a bimodal pattern (Fig. 3A), which is similar to what has been observed in somatic tissues of human and mouse^31^. Further, consistent with the reports that CpG islands are usually unmethylated^31^, the majority of the CpGs within CpG islands in our study were hypomethylated (Fig. 3A,B). In addition, we explored the relationship between the DNA methylome and the transcriptome. We first plotted the average methylation of all the annotated genes along the gene body and 3 kb upstream of the transcription start site (TSS) and 3 kb downstream of the transcription end site (TES). The 3 kb range was chosen because the average DNA methylation beyond this distance was ~0.6–0.7, which was the overall average methylation of bovine fetal skeletal muscle. As shown in Fig. 3C, we found a negative correlation (Spearman rank test, p < 0.02) between the gene expression level and the level of DNA methylation at regions near the TSS (−500bp to TSS) (Fig. 3C). Furthermore, we queried the relationship between DNA methylation and transcript abundance for genes with different levels of CpG density at their promoter regions. For genes with high (HCP) and intermediate (ICP) CpG density promoters, there was a negative correlation (Spearman rank test, p < 0.02) between the methylation level at promoter regions and the transcript abundance (Fig. 3C). However, low CpG density promoters (LCP) were usually methylated and had no significant correlation with gene expression levels (Fig. 3C). Notably, we observed a positive correlation (Spearman rank test, p < 0.02) between DNA methylation at gene bodies and transcript abundance, especially with HCP genes (Fig. 3C). Overall, in our study, the associations between global DNA methylation and global gene expression were consistent with the findings in human and mouse^31^.

### Identification and characterization of the ASM regions in bovine fetal skeletal muscle

**Figure 4:**
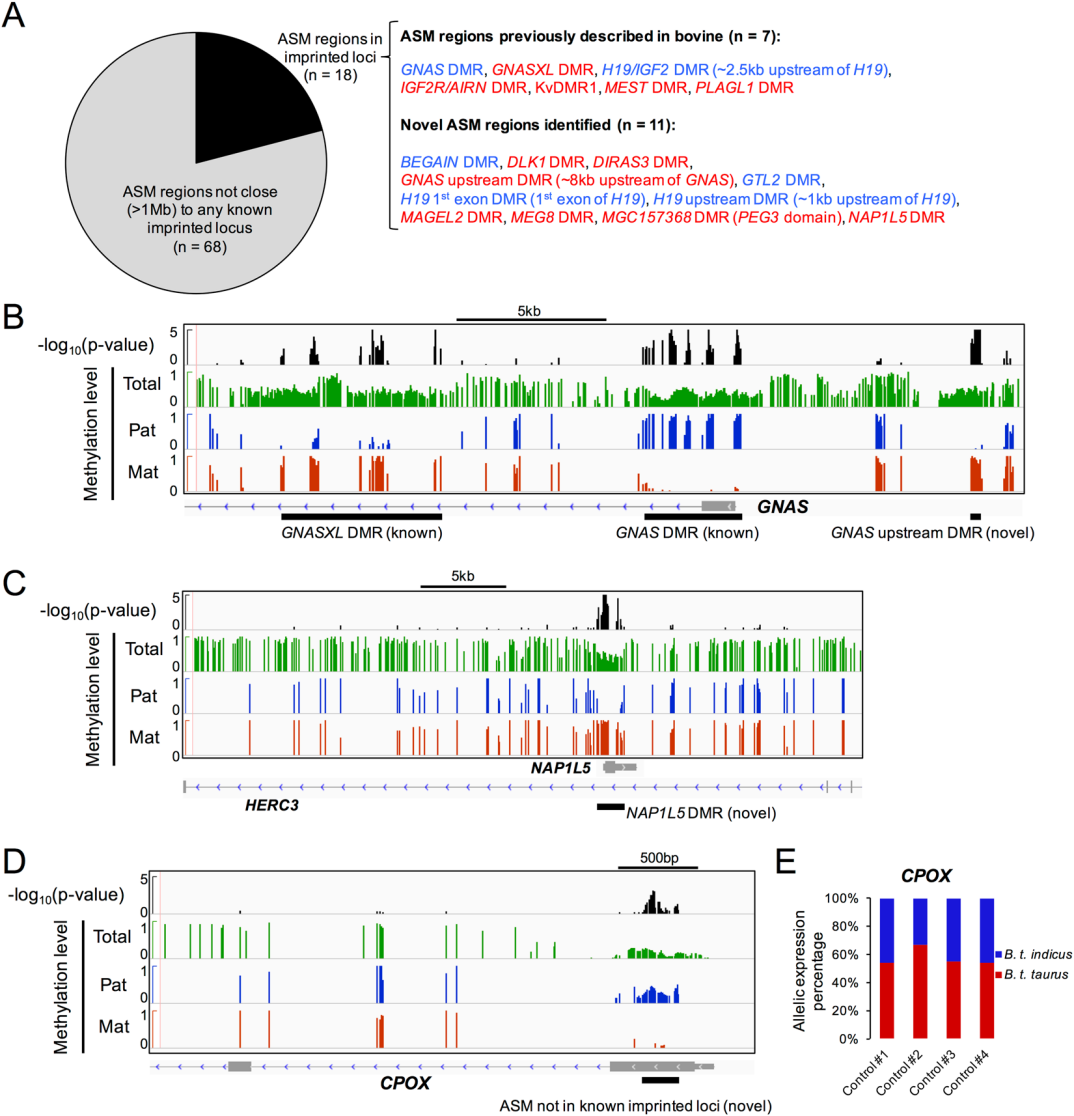
Identification and characterization of ASM regions in skeletal muscle of control fetuses. (**A**) Summary of the ASM regions identified at imprinted and non-imprinted loci. Red color: DMRs with maternal allele-specific methylation; blue color: DMRs with paternal allele-specific methylation. (**B–D**) Genome browser views of the imprinted ASM loci: *GNAS/GNASXL* and *NAP1L5/HERC3* and the non-imprinted *CPOX*. The –log_10_(p-value) for ASM significance (black) and the CpG methylation levels for total (green), *B*. *t*. *indicus* allele (blue, paternal), and *B*. *t*. *taurus* allele (red, maternal) are also shown. Each bar represents a single CpG site. (**E**) Allelic expression percentage of *CPOX* in skeletal muscle of the control fetuses. ASM: allele-specific DNA methylation; DMR: differentially methylated region. More examples may be found in Figs S5-S6.

As only a limited number of imprinted ASM regions have been characterized in bovine^5,18,32–34^, one objective of our study was to identify ASM regions at base resolution in bovine fetal skeletal muscle using WGBS. To distinguish the parental origins of the alleles in the *B*. *t*. *indicus* × *B*. *t*. *taurus* F_1_ progenies, we obtained heterozygous SNPs of the fetuses from two sources: 1) SNPs identified from the WGBS data using Bis-SNP^35^ and 2) SNPs identified from the RNAseq data of the F_1_ hybrids^33^ (Supplementary Fig. S5). We assigned the WGBS reads overlapping the SNPs to their parental origins based on the genotype of the *B*. *t*. *indicus* sire^33^ of the F_1_ fetuses. As an initial step to screen CpGs with ASM, allelic WGBS reads were combined from the four controls and CpGs with at least 4 × coverage of each allele (n = 4,798,414; 17.4% of CpGs in the bovine genome) were subject to Fisher’s exact test to determine whether DNA methylation level was significantly different between alleles. In total, 109,794 ASM candidate CpGs were identified with a p-value cutoff of 0.01 [false discovery rate (FDR) ≈ 0.05, Supplementary Fig. S5]. To further minimize the false positive ASM sites, the ASM candidate CpGs were merged into regions using a clustering method as previously described^36^. In total, 1,070 ASM CpGs were grouped into 86 discrete genomic regions (Fig. 4A and Supplementary Table S3).

We manually annotated the identified 86 ASM regions (Supplementary Table S3) and found that 18 of them were located at known imprinted loci, while others were located >1 Mb away from any known imprinted gene in bovine (Fig. 4A). The ASM regions >1 Mb from known imprinted genes are referred to as the “non-imprinted ASM” in subsequent text. The two types of ASM regions are of comparable size; however, the imprinted ASMs have a higher density of CpGs (Supplementary Fig. S6) located within CpG islands. Both imprinted and non-imprinted ASM loci have overall DNA methylation level around 0.5, but ASM regions at imprinted loci exhibit less variation (range = 0.43 [imprinted] vs 0.8 [non-imprinted] and SD = 0.11 [imprinted] vs. 0.19 [non-imprinted]; Supplementary Fig. S6). Lastly, the methylation differences between the parental alleles for the imprinted ASM regions are greater when compared to the non-imprinted ASM regions (mean = 0.73 [imprinted] vs. 0.51 [non-imprinted]; Supplementary Fig. S6).

Of the 18 imprinted ASM regions, seven have been previously described in bovine by us and others. These include the paternal DMRs: *GNAS* DMR, *H19/IGF2* DMR^33,34^ and the maternal DMRs: *GNASXL* DMR, *IGF2R/AIRN* DMR, KvDMR1, *MEST* DMR, and *PLAGL1* DMR^18,32–34^ (Fig. 4B and Supplementary Fig. S7). Of the novel imprinted ASM regions identified in bovine fetal skeletal muscle, eight of them, including *NAP1L5* DMR, *DIRAS3* DMR, *GTL2* DMR and *DLK1* DMR (Fig. 4C, Supplementary Fig. S7), have been previously reported in human and/or mouse (Supplementary Table S4). The other three novel ASM regions namely, *BEGAIN*, *MEG8*, and *MGC157368* DMRs, were located within the gene bodies of the known imprinted genes in bovine (Supplementary Table S3). All the ASM regions described above could be associated with the nearby imprinted transcriptional activities previously reported by us^33^ with the exception of *MEST* DMR, for which no heterozygous SNP was identified to enable allelic gene expression analyses.

We also manually inspected the imprinted ASM regions previously described in human and/or mouse in our bovine WGBS data. We found that five ASM regions (*i*.*e*., *GRB10* DMR, *H13* DMR, *NNAT* DMR, *PEG3* DMR, and *PEG13* DMR) were absent in this study due to either poor SNP coverage or low read coverage (Supplementary Table S4). A less stringent criterion (*i*.*e*., 3 × coverage per allele instead of 4 × ) for ASM calling could lead to the identification of another three DMRs (*i*.*e*., *INPP5F* DMR, *PEG10* DMR, and *SNRPN* DMR) (Supplementary Table S4). Lastly, we found that some imprinted DMRs in human and/or mouse were simply not subject to ASM in bovine fetal skeletal muscle as these loci were either hypomethylated (*e*.*g*., *RASGRF1* DMR, *ZRSR1/IMPACT1* DMR, and *COMMD1* DMR) or partially methylated on both parental alleles (*e*.*g*., *MKRN3* DMR and *GPR1/ZDBF2* DMR) (Supplementary Table S4).

For the 68 ASM regions not adjacent to any known imprinted locus, we sought to identify genes subject to allele-specific expression in the nearby regions. As shown in Supplementary Table S3, of the 68 ASM regions, 20 were intergenic (>5 kb away from any annotated gene) while the other 48 regions could be associated with nearby genes. For the 48 genes, parental-allele-specific read counts for 24 genes could be obtained from our previous study^33^. We found that all of these genes exhibited biallelic gene expression and had no association with the nearby ASM regions (shown in in Fig. 4D,E, and Supplementary Fig. S8 are five examples). The remaining 24 genes were either lowly expressed in skeletal muscle or had no heterozygous SNPs available to ascertain the parental alleles. It is possible that some of these ASM regions are associated with tissue-specific or transcript-isoform-specific monoallelic gene expression.

### Lack of direct associations between DMRs and DEGs in LOS

**Figure 5:**
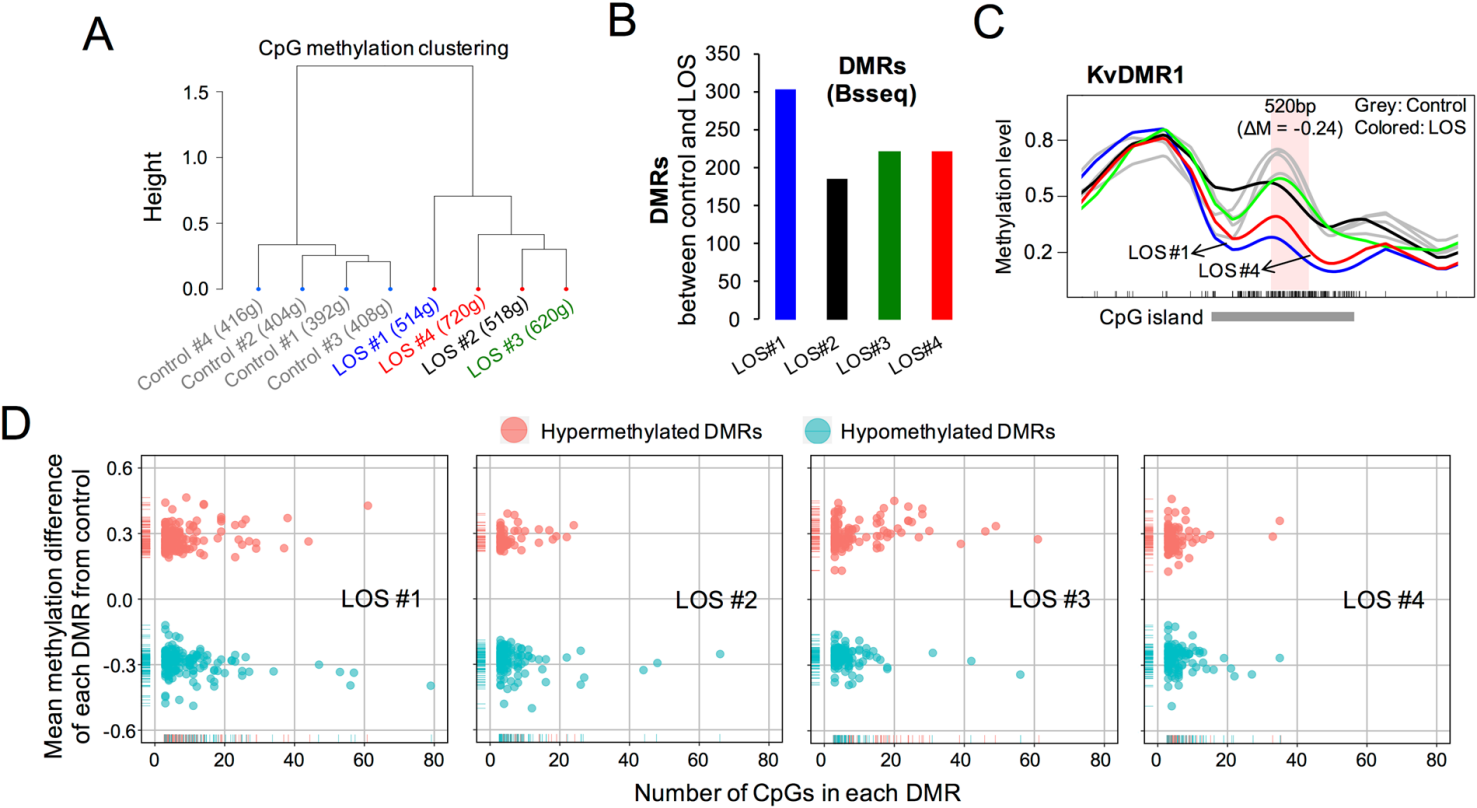
Identification and characterization of DMRs in fetal skeletal muscle between control and LOS. (**A**) Hierarchical clustering of CpG methylation within DMRs between control and LOS using 1-Pearson’s correlation distance. Bodyweight for each fetus is also shown. (**B**) Number of DMRs identified by the Bsseq R package between each LOS fetus and all four controls. (**C**) Smoothed methylation profiles of the F_1_ fetuses at KvDMR1 with the pink area representing the DMR identified (size = 520 bp; ΔM = mean methylation difference of the DMR between all four LOS and the controls; grey = controls; blue = LOS #1; black = LOS #2; green = LOS #3; red = LOS #4). Ticks at bottom represent CpG sites. (**D**) Number of CpGs and the mean methylation difference of each DMR identified in each LOS fetus.

As for the DEG analysis, each LOS fetus was analyzed independently and compared to the average of all four controls to identify DMRs using the Bsseq R package^30^. The hierarchical clustering of CpG methylation within DMRs showed that control fetuses clustered together and were separated from the LOS fetuses (Fig. 5A). The numbers of DMRs identified were similar for the four LOS fetuses with most DMRs identified in LOS #1 (Fig. 5B). Notably, we identified loss of DNA methylation of KvDMR1 in LOS#1 and #4 (Fig. 5C), indicating the validity of the statistical method used for DMR determination, as we have previously identified these specific epimutations by the bisulfite polymerase chain reaction (PCR), cloning, and sequencing^5^. However, our previous study reported a single DMR with a size of 385 bp (37 CpGs) while the WGBS analyses allowed us to identify methylation differences between control and LOS across the entire CpG island (1957bp, 207 CpGs, UMD3.1 Chr29: 49,552,845–49,554,801). Further characterization of the DMRs revealed that most of them contained less than 20 CpGs and were of methylation differences between 0.2–0.5 (Fig. 5D).

**Figure 6:**
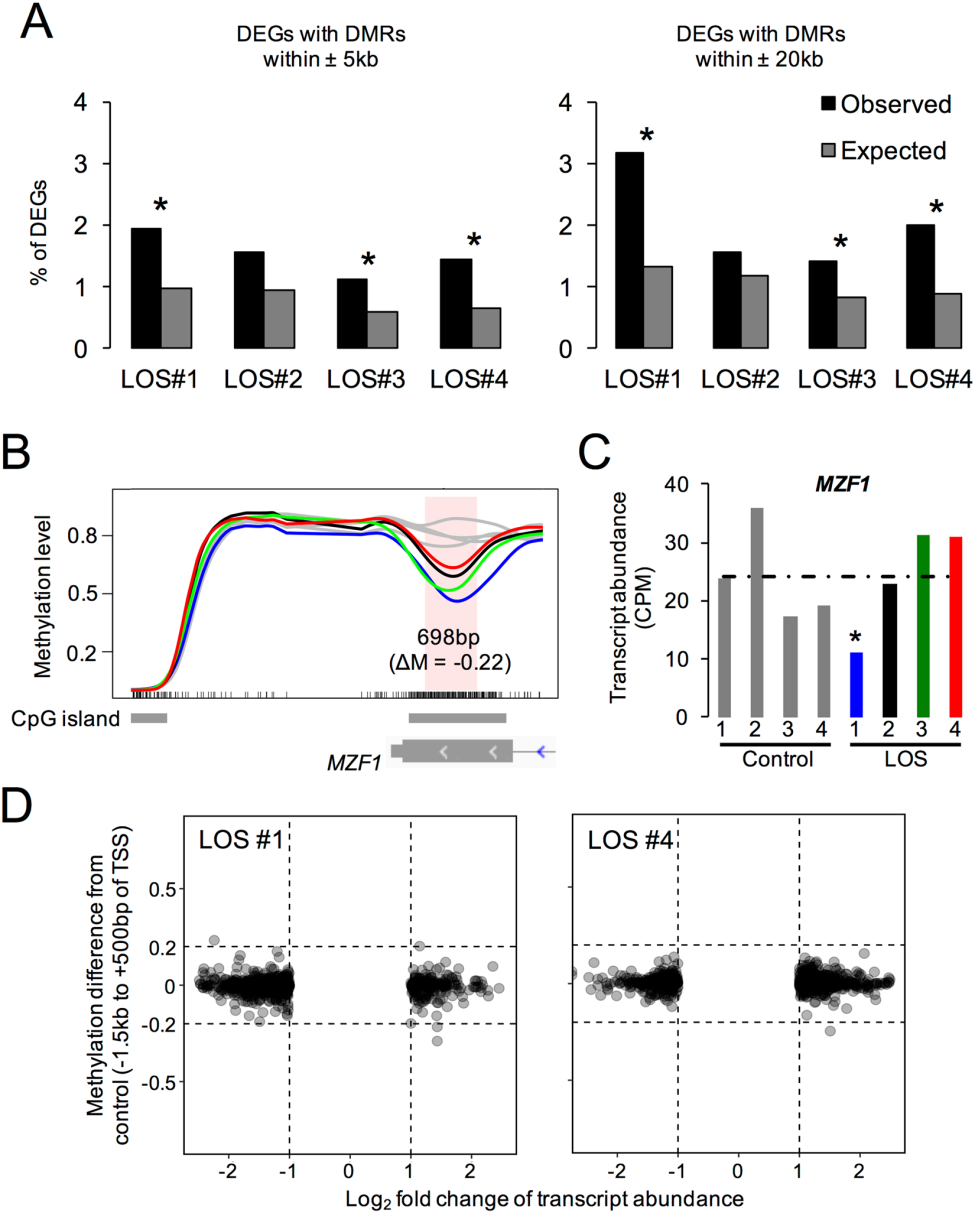
Lack of associations between the DEGs and the DMRs in skeletal muscle of the LOS fetuses. (**A**) Percentage of DEGs that are associated with the DMRs in LOS fetuses. A DMR is considered to be associated with a DEG if it is less than 5 kb or 20 kb away from the DEG. To determine whether the number of DEGs associated with DMRs were greater than expected by chance, DMRs were shuffled within each chromosome 1000 times and the expected numbers of DEGs associated with the DMRs were calculated as the average of 1000 datasets and were used in chi-square tests (*p < 0.01). (**B,C**) Examples of lack of association between DEGs and DMRs when the DMRs are within 5 kb range of the DEGs. For (**B**), the details may be found in Fig. 5. Panel (**C**) shows the transcript abundance of *MZF1* in all fetuses (asterisk: edgeR FDR < 0.05). DEG: differentially expressed genes. (**D**) Mean methylation level differences of the DEGs between control and LOS fetuses at the TSS region (−1.5 kb to + 500 bp). Only DEGs that had at least 2-fold change are shown here.

We then characterized the genomic distances between the identified DMRs and the DEGs, in order to determine whether any of the DMRs could be linked to aberrant gene expression in LOS. We first queried the genomic regions within 5 kb range of the DEGs and found that on average only ~1.5% of DEGs in each LOS fetus could be associated with the identified DMRs (Fig. 6A). Increasing the range queried from 5 kb to 20 kb only added an additional ~0.5% of DEGs that associate with the identified DMRs (Fig. 6A). The locations of the identified DMRs were randomly permutated 1000 times to determine whether the numbers of DEGs that are associated with the DMRs were greater than expected by chance. For each dataset, DEGs were considered to be associated with altered DNA methylation if DMRs were within 5 kb or 20 kb range of the DEGs. The expected counts of DEGs were calculated as the average of the 1000 randomized datasets. As shown in Fig. 6A, DEGs are associated with DMRs more often than expected by chance in LOS #1, #3, and #4 but not in LOS #2.

We found that 1407 DEGs are common between LOS #3 and #4 (the two largest LOS fetuses) and we sought to determine if differential DNA methylation can explain misexpression of these genes (Fig. 2C). Only 14 DMRs overlapped between LOS #3 and #4, and only one of these was located within 5 kb of one common DEG (*C5H12orf45*) and another within 20Kb (*RWDD1*). We also noted that not all LOS fetuses with aberrant DNA methylation at the same locus had differential gene expression of the associated gene (an example shown in Fig. 6B,C), suggesting that differential DNA methylation is not the sole cause of differential gene expression. Lastly, minor methylation differences were detected in the TSS regions (−1.5 kb to + 500 bp) of the DEGs between control and LOS (Fig. 6F and Supplementary Fig. S9). In summary, only a very small percent DEGs in fetal skeletal muscle could be directly associated with the DMRs between control and LOS.

### Association of aberrant DNA methylation and differential gene expression at *IGF2R/AIRN* and *MAGEL2* imprinted loci

**Figure 7:**
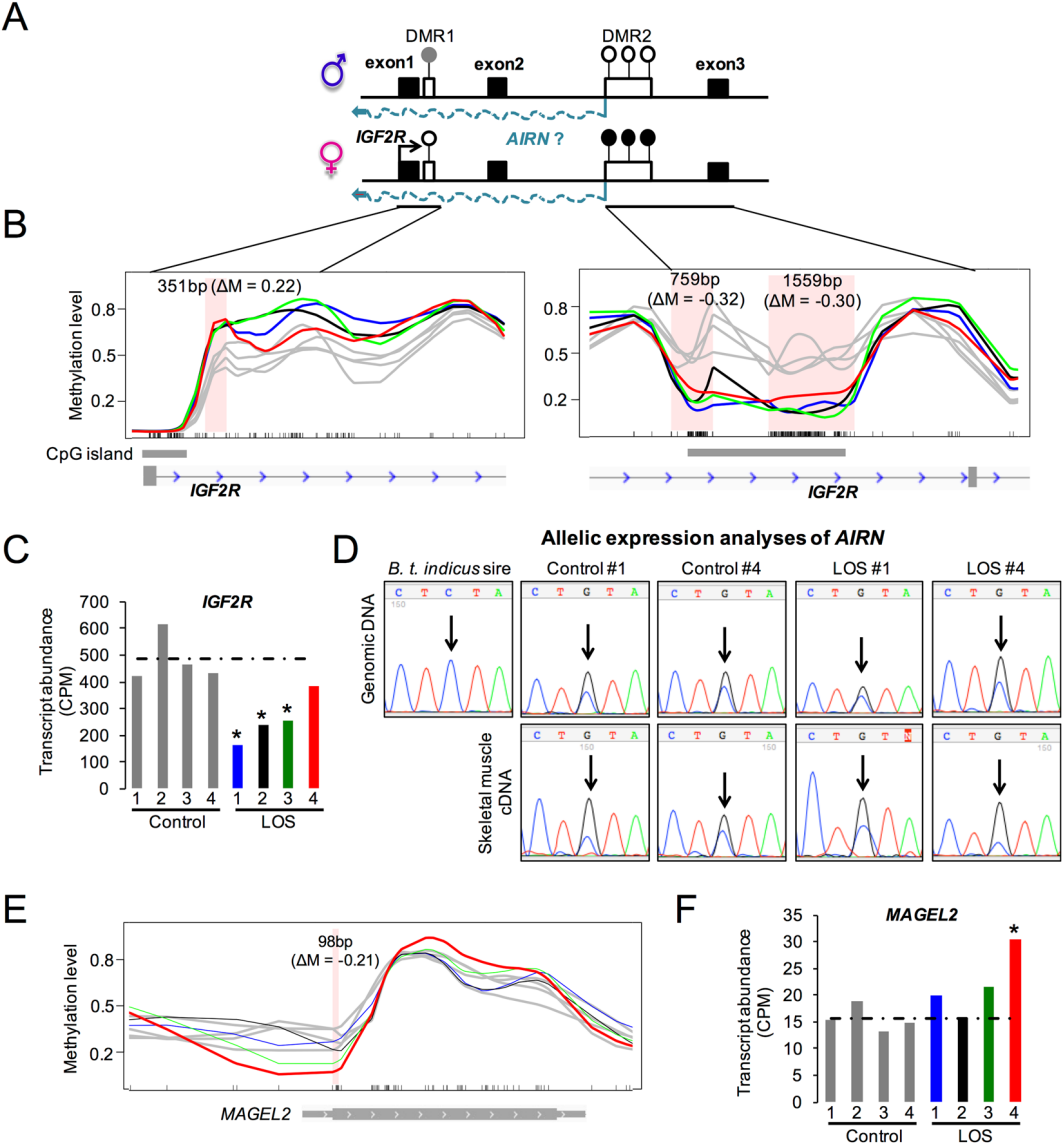
DNA methylation and differential gene expression at *IGF2R/AIRN* and *MAGEL2* imprinted loci. (**A**) Schematics of the *IGF2R/AIRN* locus. Bent arrows represent the transcription start sites. White boxes represent the ASM regions (white lollipops = unmethylated; grey lollipop = partially methylated; black lollipops = methylated). (**B**) Smoothed methylation profiles for the two DMRs at *IGF2R* locus between control and LOS fetuses. The pink areas represent the DMR identified (ΔM = mean methylation difference of the DMR between all four LOS and the controls; grey = controls; blue = LOS #1; black = LOS #2; green = LOS #3; red = LOS #4). Ticks at bottom represent CpG sites. (**C**) Transcript abundance of *IGF2R* in control and LOS fetuses (asterisk: edgeR FDR < 0.05, LOS vs control). (**D**) Allelic expression analyses for *AIRN* by Sanger sequencing. The SNP site is indicated by an arrow. (**E**) Smoothed methylation profile of *MAGEL2* locus. The labels are as panel B. (**F**) Transcript abundance of *MAGEL2* in the F_1_ fetuses (asterisk: edgeR FDR < 0.05).

Insulin-like growth factor 2 receptor (IGF2R) is a scavenger receptor of the fetal growth factor IGF2, and is maternally expressed in cow^18,37^ (Fig. 7A) and mouse^38^, but exhibits polymorphic imprinting in human^39^. In this study, *IGF2R* exhibited decreased transcript abundance in three out of four LOS fetuses (Fig. 7C), and this differential gene expression was associated with the altered DNA methylation at DMR1 and DMR2 of the *IGF2R* locus (Fig. 7A-B, Supplementary Fig. S9). In all four LOS fetuses, we found loss of methylation of DMR2, which is located in the second intron of *IGF2R* with loss of methylation in all four LOS fetuses (Fig. 7B). Normally, DMR2 was methylated on the maternal allele and unmethylated on the paternal allele. Hypomethylation of DMR2 has also been reported in sheep LOS^40^, suggesting the *IGF2R* locus is similarly misregulated in these two species. DMR1, which is close to the promoter region of *IGF2R*, is normally partially methylated on the paternal allele and unmethylated on the maternal allele. In our study, DMR1 was hypermethylated in LOS (Fig. 7A,B).

In mice, paternal silencing of *IGF2R* is mediated by the paternally expressed long non-coding RNA *AIRN*, which is transcribed in the antisense direction to *IGF2R* and is regulated by DMR2 of *IGF2R* ^41^. Thus, we reasoned that hypomethylation of DMR2 can lead to biallelic expression of *AIRN*, which represses the expression of *IGF2R* on both alleles and results in the decreased level of *IGF2R* in LOS fetuses. However, contrary to our expectation, *AIRN* was biallelically expressed in both control and LOS fetal skeletal muscle (Fig. 7D), suggesting that down-regulation of *IGF2R* is not mediated by biallelic expression of *AIRN*. Nevertheless, decreased transcript abundance of *IGF2R* was associated with a hypomethylated DMR2 and hypermethylated DMR1 in LOS fetuses.

In addition to *IGF2R*, we detected an association between differential gene expression and differential DNA methylation at the *MAGEL2* locus. *MAGEL2* is a paternally expressed gene^18^ with its promoter methylated on the maternal allele but unmethylated on the paternal allele (Supplementary Table S3). As shown in Fig. 7E,F, the increased transcript abundance of *MAGEL2* in LOS #4 is associated with the loss of DNA methylation at its promoter region.

## Discussion

Since BWS was first described in the early 1960s^42^ and the first cases of ART-induced LOS were reported in 1995^43^, the main focus in both fields has been to understand how loss-of-imprinting alone can contribute to the complex and variable phenotypic features of these overgrowth conditions. In human, the epimutation and genetic alteration of the two imprinted loci (*i*.*e*., *KCNQ1* and *H19/IGF2* loci) affect nearly 70% of BWS patients with the hypomethylation at KvDMR1 accounting for ~50% of all BWS cases^19^. In addition, loss-of-imprinting at multiple loci was observed in ~25% of BWS cases with epimutation at the *KCNQ1* locus^19^. Similarly in bovine, we have also shown that LOS exhibits loss-of-imprinting at multiple loci including the *KCNQ1* locus^5,18^. However, to date, there has been no report of molecular features that can consistently and reliably diagnose and predict these syndromes and the associated variable phenotypes. Here we advance the field by describing the altered transcript abundance of genes beyond imprinted loci in LOS fetuses, showing that loss-of-imprinting is not be the only molecular lesion of this condition.

In this study, WGCNA and KEGG pathway analyses identified that multiple pathways involved in myoblast proliferation and fusion of myoblasts into myotubes are misregulated in skeletal muscle of LOS fetuses. This finding is consistent with the previous report that short-term culture of ovine embryos can affect myogenic programming and the altered myogenesis may contribute to the large muscles observed in the oversized fetuses^44^. In addition to skeletal muscle, altered transcript abundance of genes was also detected in liver, brain, and kidney of the LOS fetuses. Interestingly, tissue specific alterations in transcript abundance varied between LOS fetuses. For example, LOS #1 had the most DEGs in liver, LOS #3 in kidney and LOS #4 in muscle. This observation is supported by findings in BWS in which different individuals have different susceptibility for Wilms tumor of the kidney or hepatoblastoma^19^. Future studies with in depth characterization of the histology and the transcriptome of the tissues at various developmental stages of LOS will better elucidate relationships between the altered gene pathways and the potential disturbed development of these organs.

An interesting finding of this study was that only a small percent of DEGs could be linked to the identified DMRs between control and LOS. Recently, it has been shown that histone modifications such as H3K27me3 at distal regions in oocytes can escape epigenetic reprogramming and maintain inheritance during the pre-implantation development^45^. It is possible that molecular marks like H3K27me3 in bovine may act as an epigenetic memory of the use of ART procedures and mediate the differential gene expression and the aberrant development in later stages. Alternatively, the DEGs identified in this study could represent the downstream effect of the aberrant expression of developmentally important genes affected by ART during pre-implantation development. Lastly, it is also possible that some of the DEGs identified in our study can be attributed to germplasm interaction(s) between *B. t. indicus* and *B. t. taurus* subspecies. In summary, our study demonstrates that altered transcriptome in LOS skeletal muscle is largely uncoupled to DNA methylome epimutations and we submit that caution should be exercised in interpreting results from DNA methylation data alone when studying the etiology of these overgrowth syndromes.

In addition to providing novel insights on the etiology of LOS, our study characterized the cytosine methylation landscape at base resolution in bovine. Consistent to what has been previously reported for other mammals, CpG islands in bovine are hypomethylated and a negative association between DNA methylation and gene expression levels are observed at HCP and ICP, but not LCP promoters^31^. Furthermore, we have identified and characterized novel ASM at both imprinted and non-imprinted loci in bovine. For the imprinted loci, we have expanded the number of imprinted ASM in bovine by describing several novel DMRs such as *DIRAS3* DMR, *GTL2* DMR, and *NAP1L5* DMR. Moreover, we have shown that some known imprinted DMRs in human and/or mouse (*e*.*g*., the *L3MBTL* DMR in human and the primary DMRs *RASGRF1* and *IMPACT* in mouse) are not subject to ASM in fetal bovine skeletal muscle, suggesting species-specific imprinting of these genes. Lastly, we have identified ASM regions at non-imprinted loci which others^46^ have proposed may be the result of *cis-*acting regulatory polymorphisms. It should be noted that ASM regions identified in this study are not comprehensive because we could not assign all WGBS reads covering CpGs to either parental alleles due to the lack of informative SNPs. In future studies, analyses of the F_1_ hybrids of different crosses may further improve the identification of ASM CpG sites.

In conclusion, we have shown that misregulation of non-imprinted genes in addition to loss-of-imprinting characterizes to the ART-induced overgrowth syndrome and have demonstrated that most of the aberrant gene expression in fetal skeletal muscle is not directly associated with aberrant DNA methylation.

## Materials and Methods

An expanded and detailed version of the methodology can be found in the Supplementary file.

### RNAseq data analyses

The production of the *Bos taurus indicus* × *Bos taurus taurus* (*B*. *t*. *indicus* × *B*. *t*. *taurus*) F_1_ fetuses and collection of the fetal tissues were previously described by us^18^. Briefly, for control fetuses, synchronized *B*. *t*. *taurus* (Holstein breed) females were artificially inseminated with semen from one *B*. *t*. *indicus* bull (Nelore breed; ABS CSS MR N OB 425/1 677344 29NE0001 97155) and the F_1_ fetuses were collected via caesarean section at day ~105 of gestation (104–106). For *in vitro* produced fetuses, *B*. *t*. *taurus* cumulus-oocyte complexes were matured and fertilized *in vitro* with the same sire as for controls and embryos that developed to the blastocyst stage were transferred into synchronized *B*. *t*. *taurus* recipients. On day ~105, conceptuses were collected as described for the control fetuses. All animal procedures were performed at TransOva Genetics by veterinarians, and all procedures were approved by their animal care and use committee (Protocol number – MRP2010–001) and were conducted in a manner conforming to Trans Ova Genetics policies and procedures and the Guide for the Care and Use of Laboratory Animals. RNAseq data of the control and LOS fetuses were obtained from one of our previous studies and were processed as previously described^18,33^. Briefly, RNAseq reads were subjected to adaptor removal and quality trimming prior to alignment to the genome. To minimize single reference genome alignment bias^47,48^, HISAT2 (version 2.0.0)^49^ was used to align RNAseq reads to a diploid genome: the *B*. *t*. *taurus* reference genome (UMD3.1) and a pseudo *B*. *t*. *indicus* genome^33^. The alignment of the RNAseq reads to the diploid genome was then merged to identify: i) the RNAseq reads that were aligned to the same single genomic position in both the *B*. *t*. *taurus* reference genome and the pseudo *B*. *t*. *indicus* genome and ii) reads that were uniquely aligned to either genome but not aligned to the other. DEGs between each LOS fetus and the mean of all four controls were determined with the consideration of variance among the controls using the edgeR package^21,22^. In addition, RNAseq read length was included as a covariate for edgeR analyses as the RNAseq reads of liver, skeletal muscle, and brain from Control#2 were sequenced with a different read length compared to other samples (50 bp vs. 100 bp). The WGCNA package^24^ was used to construct a weighted gene co-expression network for the skeletal muscle of the four control and four LOS fetuses. Further, the identified network modules were correlated with the bodyweight to identify genes in modules most likely responsible for the altered bodyweight. In addition, KEGG pathway analyses for the significant network modules and the DEGs in LOS fetuses were performed using the Database for Annotation, Visualization and Integrated Discovery (DAVID)^23^.

### WGBS library preparation and sequencing

Genomic DNA isolated from skeletal muscle of four control and four LOS day ~105 *B*. *t*. *taurus* × *B*. *t*. *taurus* F_1_ female fetuses^18^ were subject to WGBS analyses. The WGBS libraries were generated using the NEBNext Ultra DNA library Prep Kit for Illumina (NEB E7370) as per the manufacturer’s instructions. Prior to sodium bisulfite mutagenesis, 0.5% unmethylated Lambda DNA was added to the bovine DNA to act as an internal control to monitor the bisulfite conversion rate. Each WGBS library was sequenced using Illumina sequencing technology to generate 100 bp paired-end reads. The raw FASTQ files are publically available at Gene Expression Omnibus (GEO accession no. GSE93775).

### WGBS data analyses

Following adaptor removal and quality trimming, WGBS read pairs were aligned to the bovine reference genome assembly UMD3.1 using Bismark (version: 0.15.0)^50^ with the default parameters. Only uniquely aligned reads were used for subsequent analyses. In order to distinguish the C > T SNPs from C > T substitutions that were caused by sodium bisulfite conversion in the F_1_ fetuses, Bis-SNP (version: 0.82.2)^35^ was used to perform the SNP calling. Only CpG sites that showed consensus CpG context for both parental alleles (*i*.*e*., do not overlap any SNPs identified by Bis-SNP) were used for the subsequent analyses. The methylation level was determined using the reads from both forward and reverse strands covering the same symmetric CpG site. The methylation level was calculated as: (number of “C” reads)/(number of “C” reads + number of “T” reads).

In order to identify ASM regions, the WGBS read pairs overlapping SNPs were assigned to their parental origin based on the genotype of the *B*. *t*. *indicus* sire^33^. Further, the allelic WGBS reads were pooled from four controls to determine the sequencing depth and only CpGs that had at least 4 × coverage of each allele were used for Fisher’s exact test. To estimate the FDR, WGBS read pairs overlapping SNPs were randomly assigned to be either *B*. *t*. *indicus* or *B*. *t*. *taurus* in origin in order to generate a randomized dataset of ASM sites^36^. As an initial screen, ASM candidate CpGs were identified using a p-value of 0.01, resulting in 109,794 ASM sites with a FDR of ~5% (compared to the 5507 CpGs identified as ASM sites in the permutated datasets). To minimize false positives, the identified ASM candidate sites were clustered into regions as previously described^36^. Methylkit R package^51^ was used to perform the hierarchical clustering of the methylation profiles. The DMRs between each LOS fetus and all four controls were identified using the Bsseq R package^30^.

To estimate the expected number of DEGs associated with DMRs, the identified DMRs were randomly shuffled within each chromosome using “shuffleBed” function from BedTools^52^. The UMD3.1 genome assembly gaps from UCSC genome Browser were excluded from the potential shuffled positions. Shuffling was performed 1000 times and for each dataset, a DEG was considered to be associated with a DMR if the DMR falls into the DEG coordinates that had been extended by 5 kb or 20 kb. The averaged DEG counts of the 1000 datasets were used as the expected count for chi-square test.

### Statistical analyses and data visualization

Statistical analyses were implemented with R (http://www.r-project.org/). Heat maps were generated with the R function “heatmap.2”. The averaged methylation level of the annotated genes were generated using deepTools^53^. For ASM regions, bed files were converted to the bigwig format and visualized as custom tracks in the Integrative Genomics Viewer (IGV)^54^.

### Allelic expression analyses of *AIRN*

Both DNA and cDNA of the F_1_ fetuses and DNA of the sire were amplified by PCR followed by Sanger sequencing to ascribe the parental origin of the transcript. Nucleic acid isolation, cDNA synthesis, and PCR amplifications were as previously reported^18^.

## Electronic supplementary material

Supplementary information
