# Supplementary material for: Global misregulation of genes largely uncoupled to DNA methylome epimutations characterizes a congenital overgrowth syndrome
Source: Sci Rep. 2017 Oct 4;7:12667. doi: 10.1038/s41598-017-13012-z (PMC5627257; doi:10.1038/s41598-017-13012-z)
Supplement: Supplementary file 1 — Supplementary information [file 41598_2017_13012_MOESM1_ESM.pdf]

Supplementary information

Global misregulation of genes largely uncoupled to DNA methylome epimutations characterizes a congenital overgrowth syndrome

Zhiyuan Chen, Darren Hagen, Tieming Ji, Christine Elsik, Rocío M. Rivera

Figure S1. (Related to Figure 1)

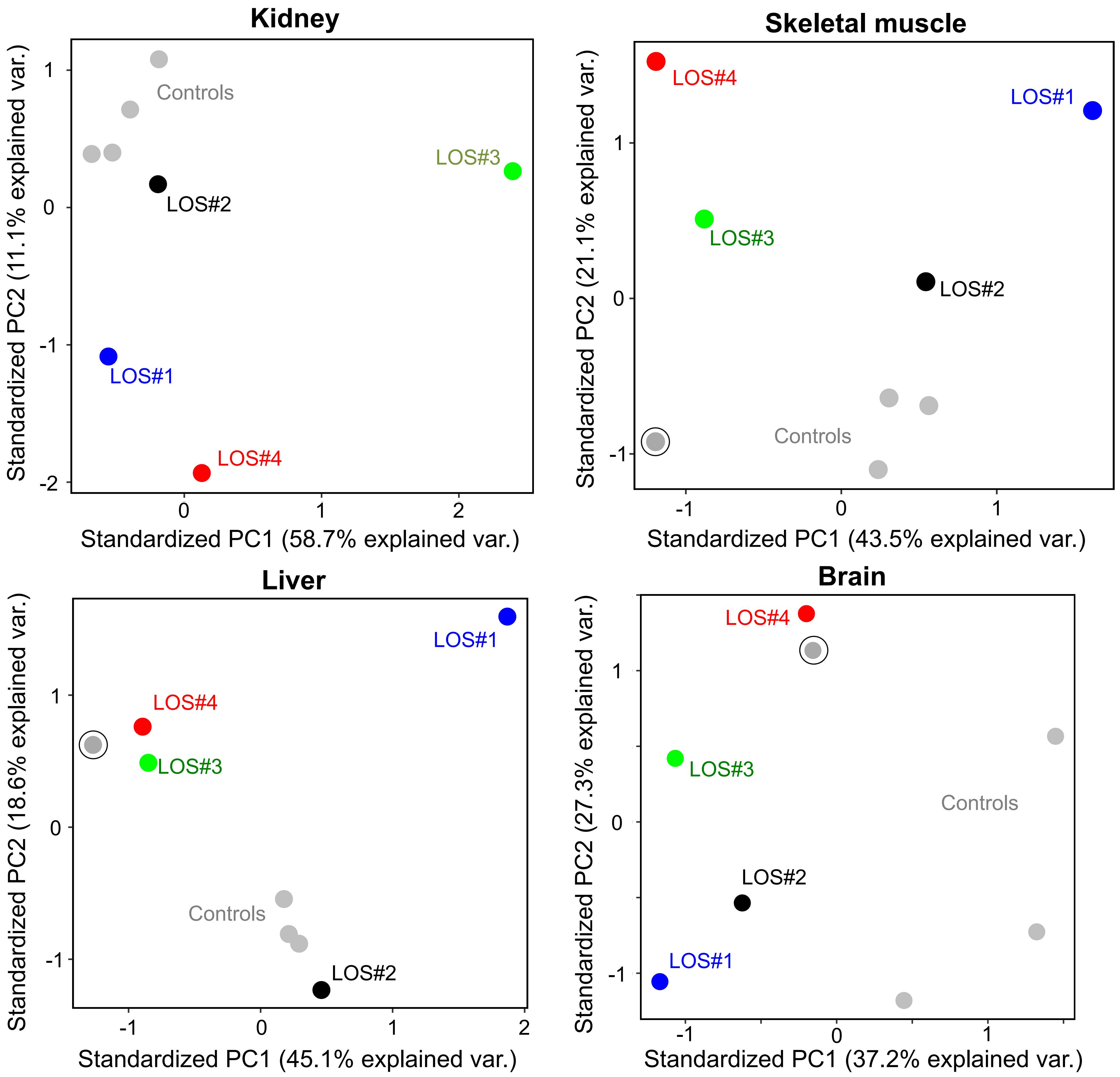

Figure S1. Principle component analyses using all expressed genes in kidney, skeletal muscle, liver, and brain. In skeletal muscle, liver, and brain, RNAseq library of Control #2 (the grey dot circled with black) was sequenced with 50bp read length, while other fetuses were sequenced with 100bp read length. The read length differences may explain why Control #2 fetus is not closely clustered with other control fetuses in these tissues.

Figure S2. (Related to Figure 1).

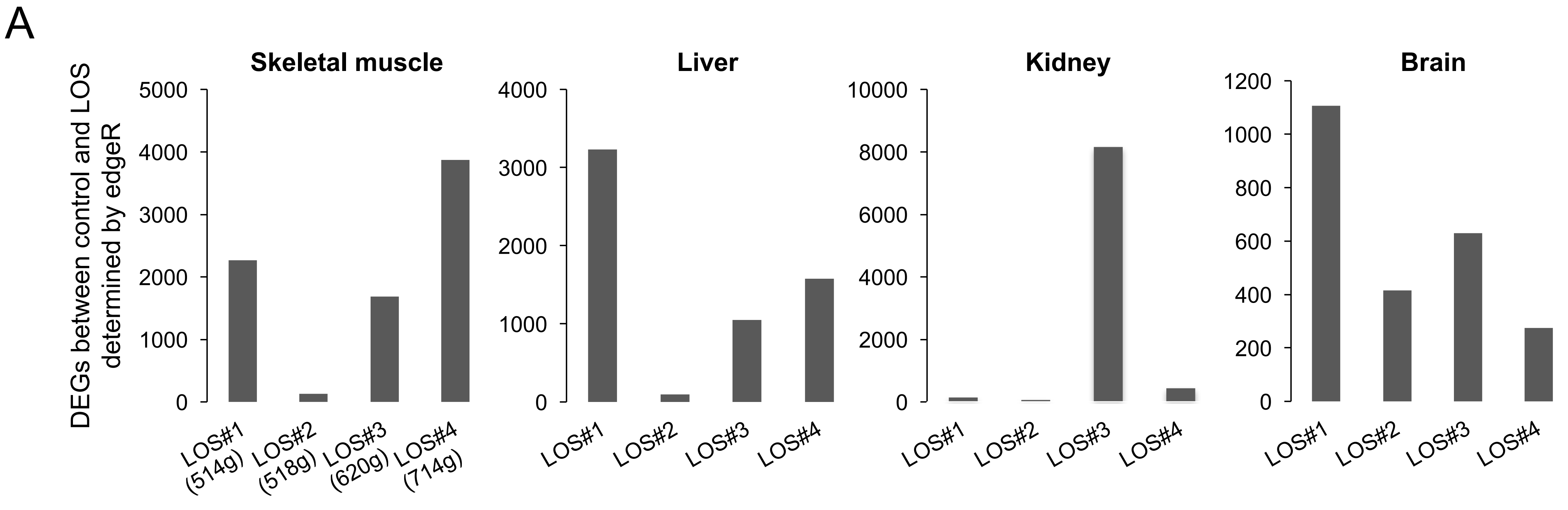

Panel B and C can be found in next page.

Figure S2. Identification and characterization of DEGs in the LOS fetuses. A). Differentially expressed genes identified between each LOS fetus and the mean of the four controls. **B).** KEGG pathways enriched for DEGs in kidney and brain (FDR < 0.05). **C).** KEGG pathways enriched for upregulated and downregulated genes in kidney and brain (FDR < 0.05).

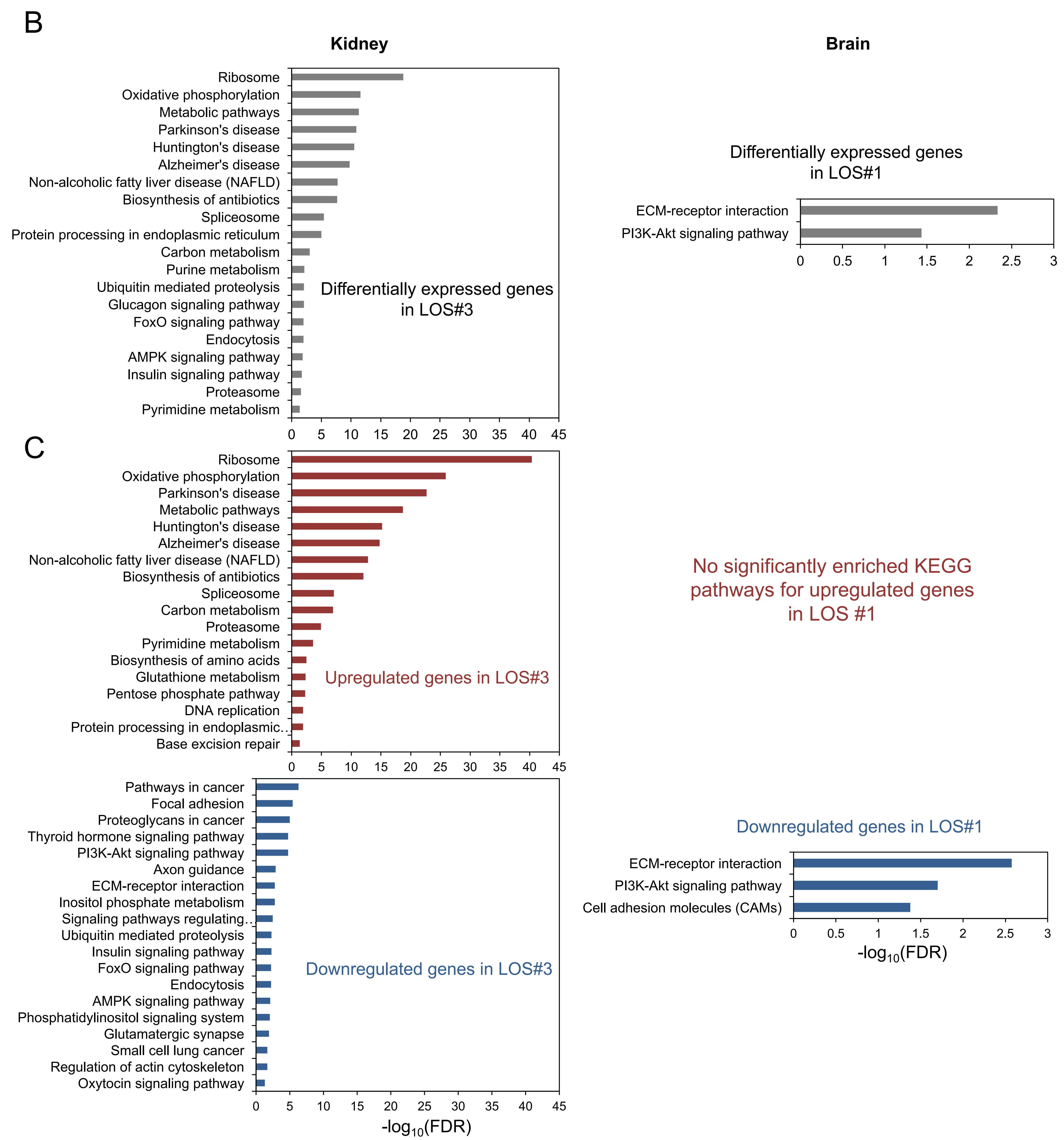
B
Kidney
Brain
Ribosome
Oxidative phosphorylation
Metabolic pathways
Parkinson's disease
Huntington's disease
Alzheimer's disease
Non-alcoholic fatty liver disease (NAFLD)
Biosynthesis of antibiotics
Spliceosome
Protein processing in endoplasmic reticulum
Carbon metabolism
Purine metabolism
Ubiquitin mediated proteolysis
Glucagon signaling pathway
FoxO signaling pathway
Endocytosis
AMPK signaling pathway
Insulin signaling pathway
Proteasome
Pyrimidine metabolism
Differentially expressed genes in LOS#3
0 5 10 15 20 25 30 35 40 45
Differentially expressed genes in LOS#1
ECM-receptor interaction
PI3K-Akt signaling pathway
0 0.5 1 1.5 2 2.5 3
C
Ribosome
Oxidative phosphorylation
Parkinson's disease
Metabolic pathways
Huntington's disease
Alzheimer's disease
Non-alcoholic fatty liver disease (NAFLD)
Biosynthesis of antibiotics
Spliceosome
Carbon metabolism
Proteasome
Pyrimidine metabolism
Biosynthesis of amino acids
Glutathione metabolism
Pentose phosphate pathway
DNA replication
Protein processing in endoplasmic…
Base excision repair
Upregulated genes in LOS#3
0 5 10 15 20 25 30 35 40 45
No significantly enriched KEGG pathways for upregulated genes in LOS #1
Pathways in cancer
Focal adhesion
Proteoglycans in cancer
Thyroid hormone signaling pathway
PI3K-Akt signaling pathway
Axon guidance
ECM-receptor interaction
Inositol phosphate metabolism
Signaling pathways regulating…
Ubiquitin mediated proteolysis
Insulin signaling pathway
FoxO signaling pathway
Endocytosis
AMPK signaling pathway
Phosphatidylinositol signaling system
Glutamatergic synapse
Small cell lung cancer
Regulation of actin cytoskeleton
Oxytocin signaling pathway
Downregulated genes in LOS#3
0 5 10 15 20 25 30 35 40 45
-log10(FDR)
Downregulated genes in LOS#1
ECM-receptor interaction
PI3K-Akt signaling pathway
Cell adhesion molecules (CAMs)
0 0.5 1 1.5 2 2.5 3
-log10(FDR)

Figure S3. (Related to Figure 2).

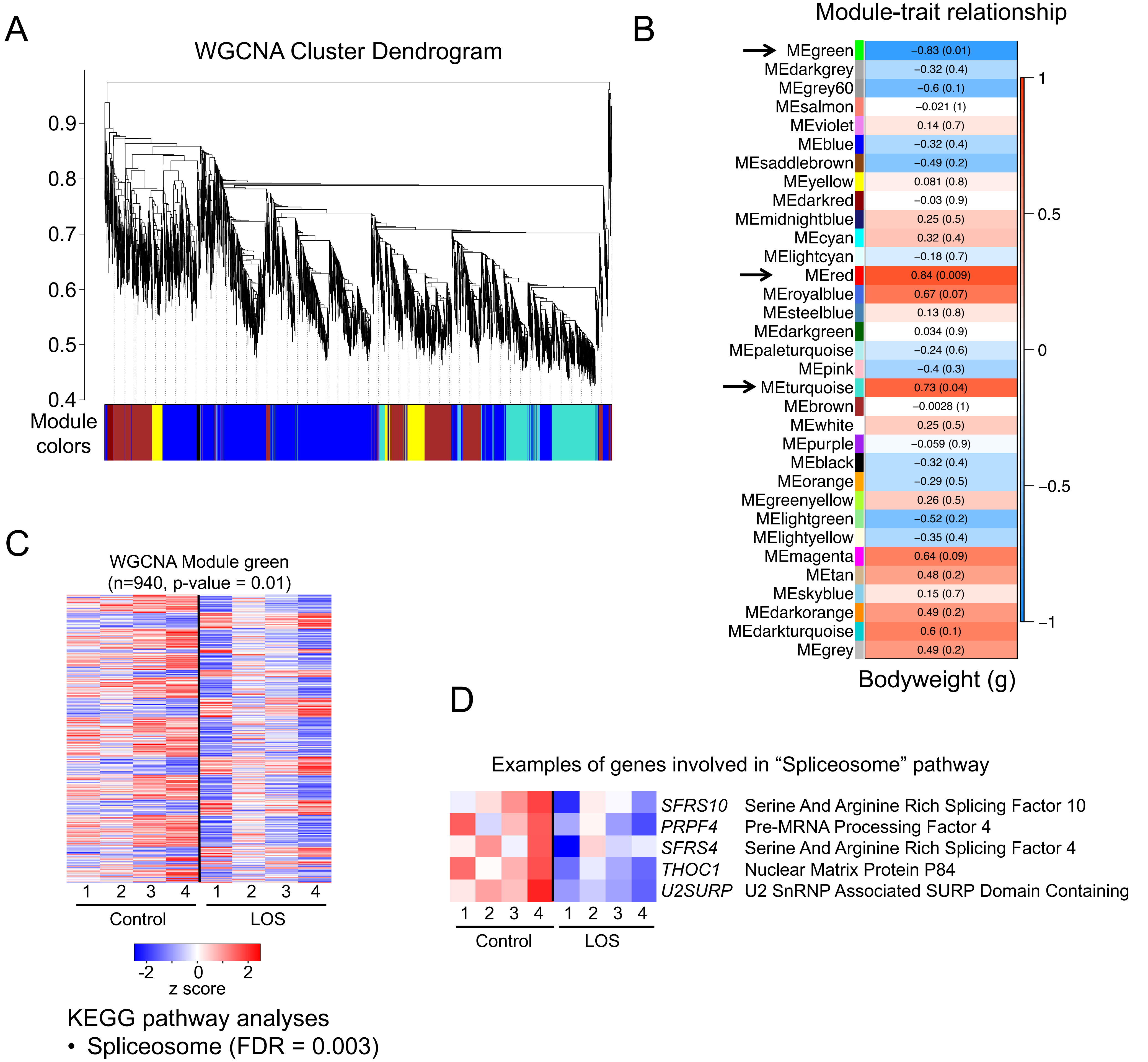

Figure S3. WGCNA and KEGG pathway analyses in skeletal muscle. A). WGCNA cluster dendrogram on all control and LOS fetuses groups genes into 33 modules, namely, 33 co-expressed gene networks. **B).** Correlation of WGCNA modules and the bodyweight of the fetuses. Arrows indicate the three modules that show significant correlation with the bodyweight. **C).** Heat map profiling of the genes of module green . Module green includes genes that are mostly misregulated in LOS #1 and #4. **D).** Heat map profiling of genes that belong to the “Spliceosome” pathway.

Figure S4. (Related to Figure 3).

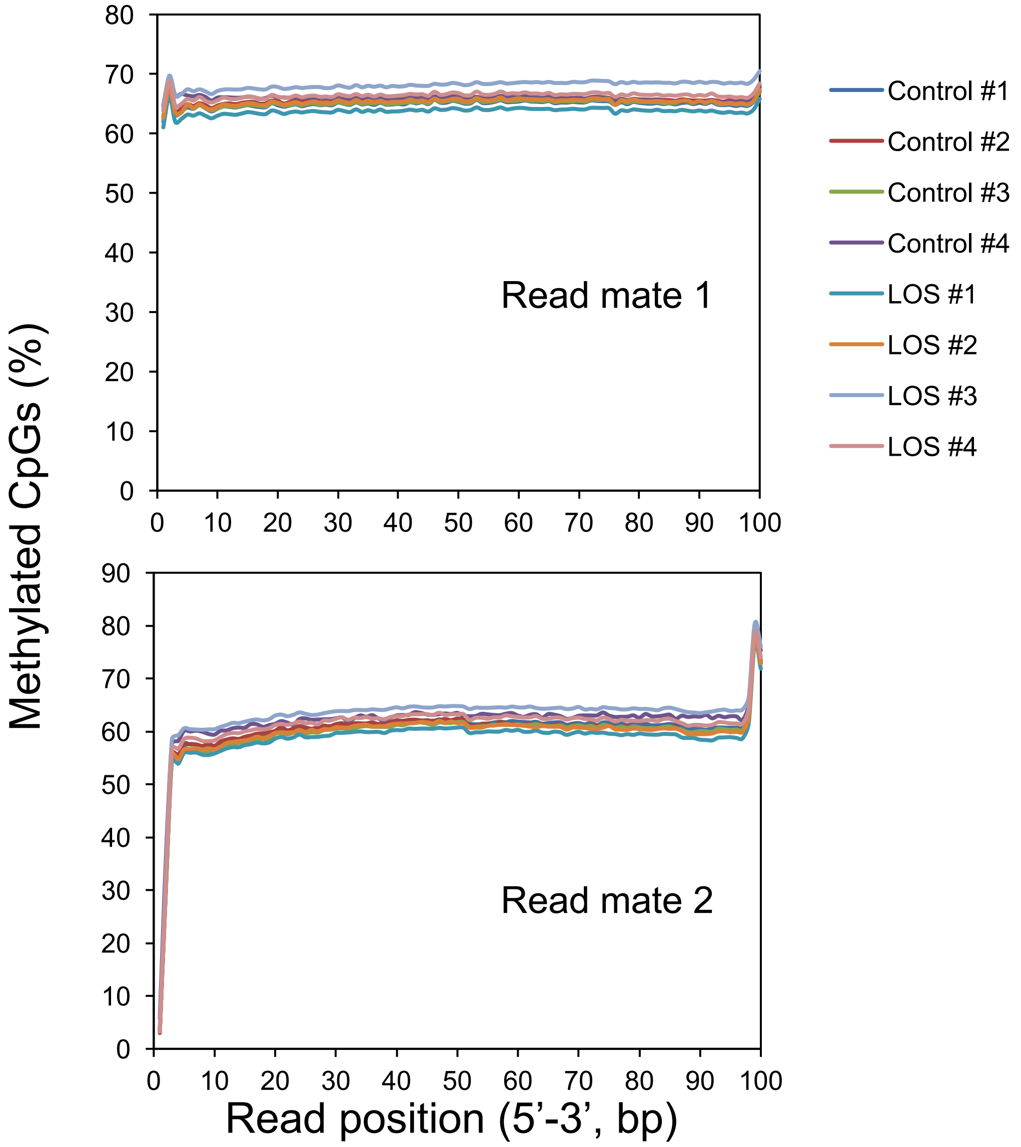

Figure S4. Means CpG methylation level at each WGBS read position of the skeletal muscle from control and LOS fetuses.

Figure S5. (Related to Figure 4).

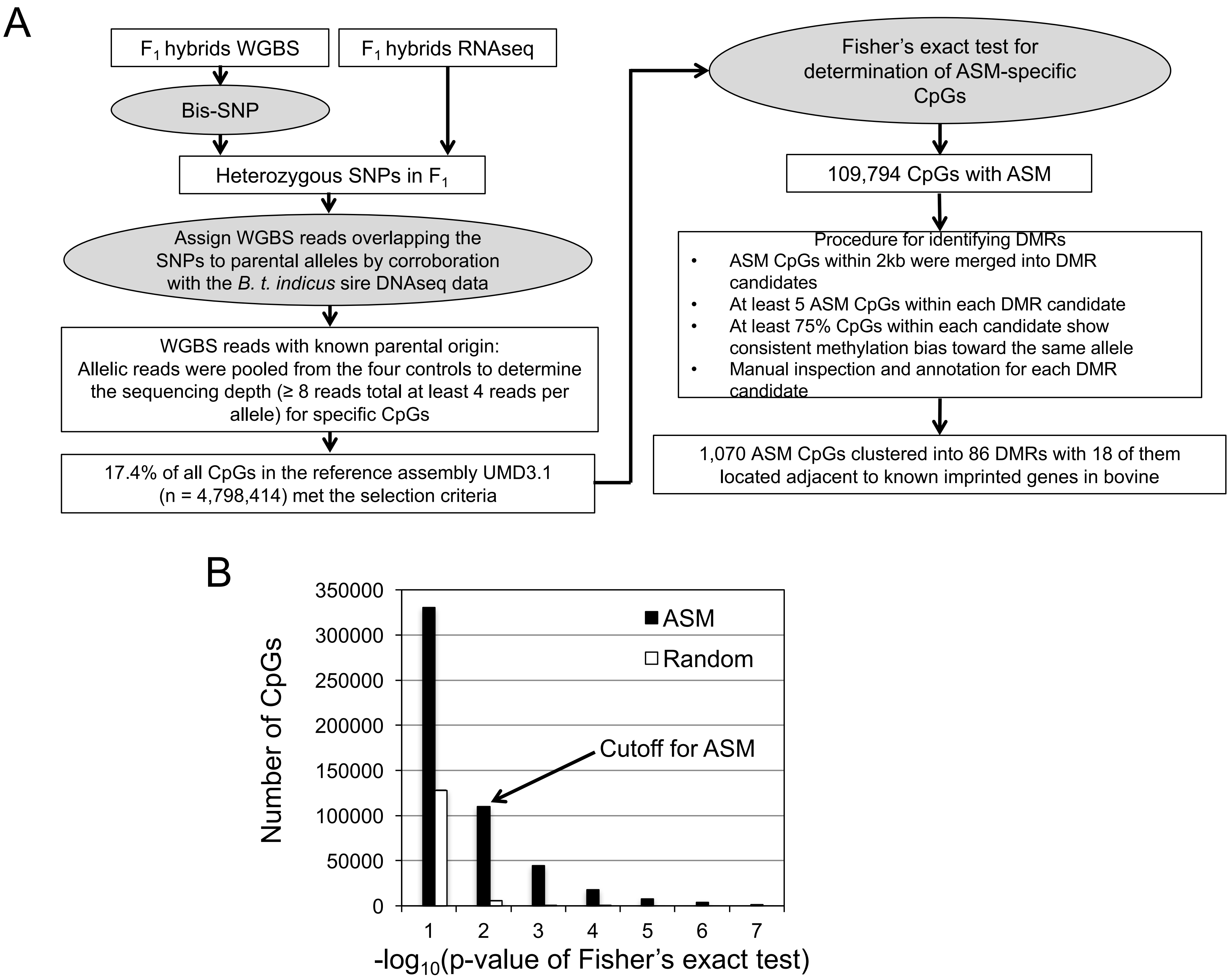

Figure S5. Pipeline used for the identification of ASM regions in skeletal muscle of control fetuses. A). Schematics pipeline. **B).** The number of ASM sites identified with various p-value cutoffs is plotted. The optimal cutoff choosed for identifying ASM sites is indicated.

Figure S6. (Related to Figure 4).

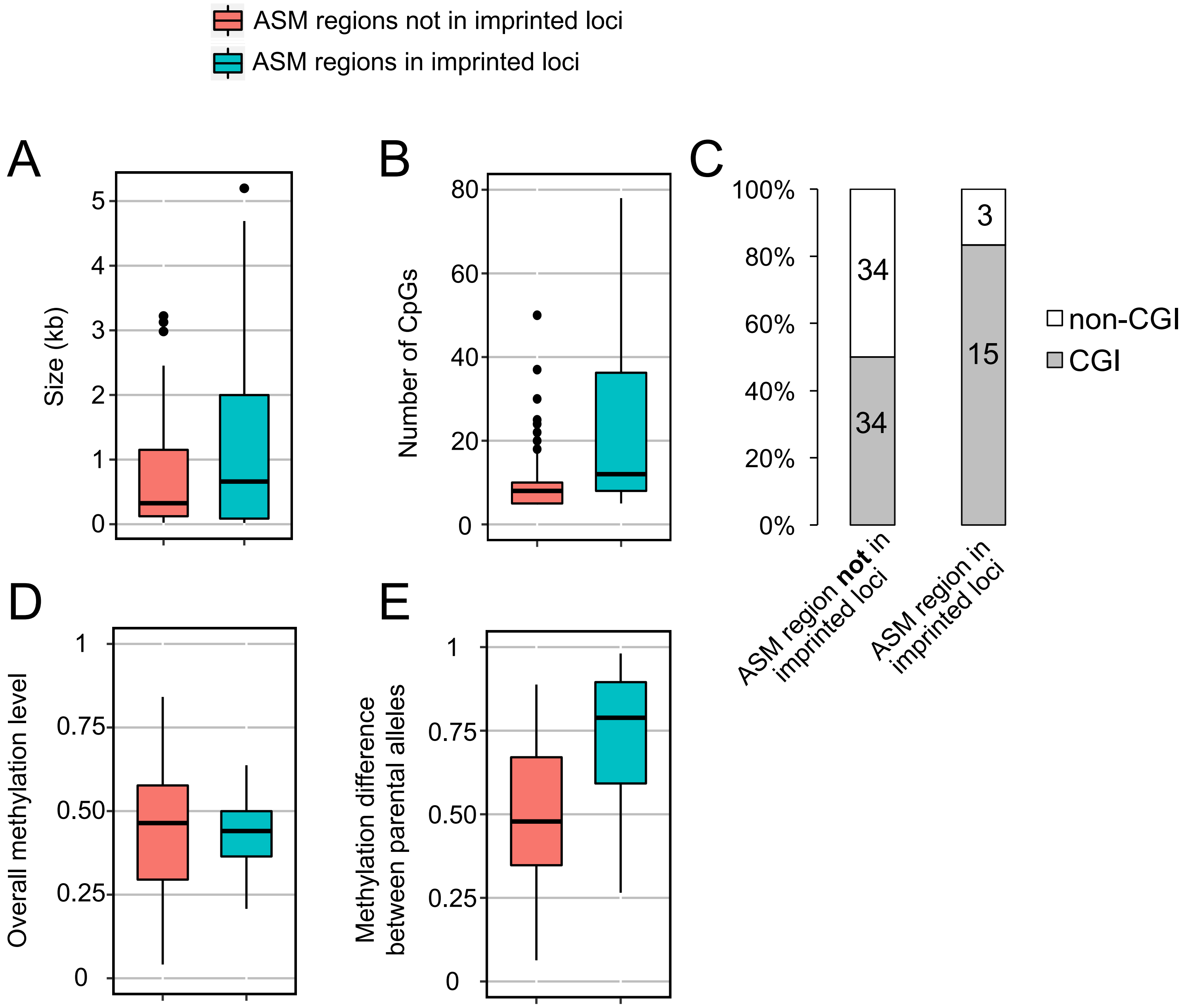

Figure S6. Characterization of the ASM regions identified in bovine fetal skeletal muscle. A) The size of the ASM regions. **B)** The number of CpGs in the ASM regions. Only CpGs identified with allele-specific DNA methylation were counted for each ASM region. **C)** Localization of the ASM regions in CpG islands. **D)** Overall methylation level for the ASM regions. **E)** Methylation differences between parental alleles for the ASM regions. CGI: CpG island.

Figure S7. (Related to Figure 4).

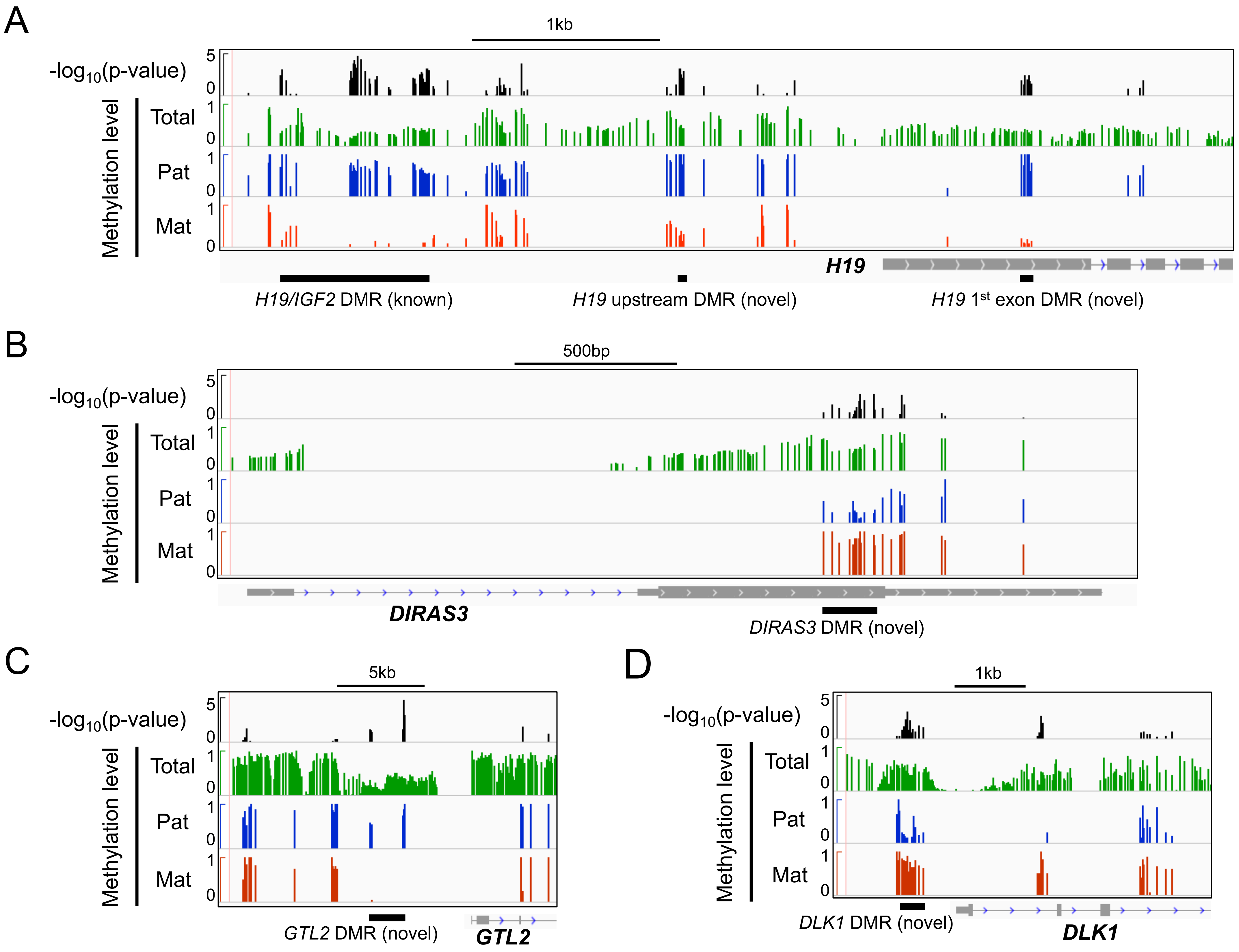

Figure S7. Examples of imprinted ASM loci. Genome browser views of the imprinted ASM loci: *H19/IGF2, DIRAS3,* and *GTL2/DLK1*. The $-\log_{10}$(p-value) for ASM significance (black) and the CpG methylation levels for total (green), *B. t. indicus* allele (blue, paternal), and *B. t. taurus* allele (red, maternal) are also shown. Each bar represents a single CpG site. Pat: paternal allele; Mat: maternal allele.

Figure S8. (Related to Figure 4).

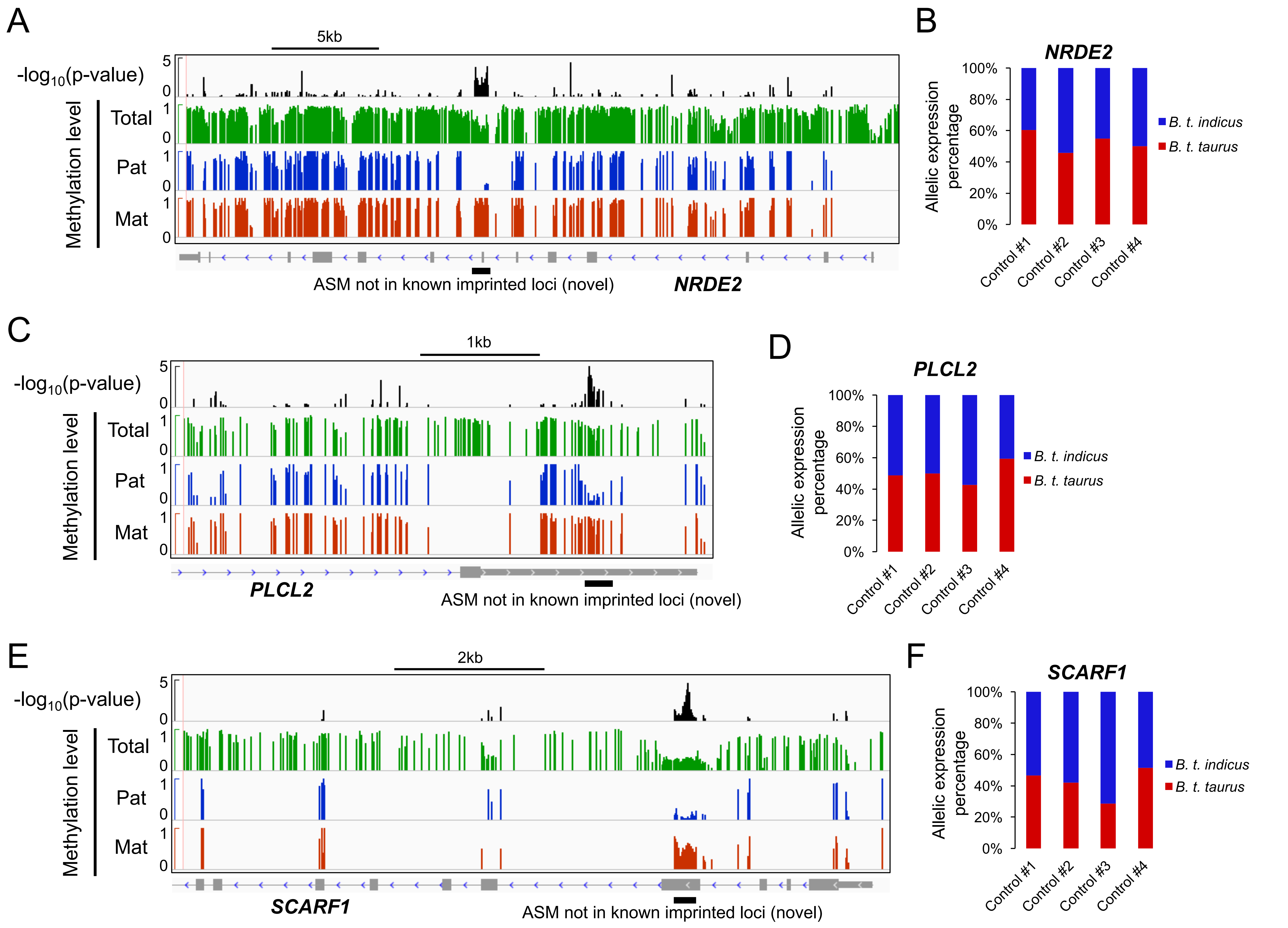

Figure S8. Examples of ASM regions at non-imprinted loci. A), C), and E). Genome browser views of the non-imprinted ASM loci: *NRDE2, PLCL2,* and *SCARF1*. The $-\log_{10}$(p-value) for ASM significance (black) and the CpG methylation levels for total (green), *B. t. indicus* allele (blue, paternal), and *B. t. taurus* allele (red, maternal) are also shown. Each bar represents a single CpG site. **B), D), and F).** Allelic expression percentage in skeletal muscle of the control fetuses. Mat: maternal allele; Pat: paternal allele.

Figure S9. (Related to Figure 5 and 6).

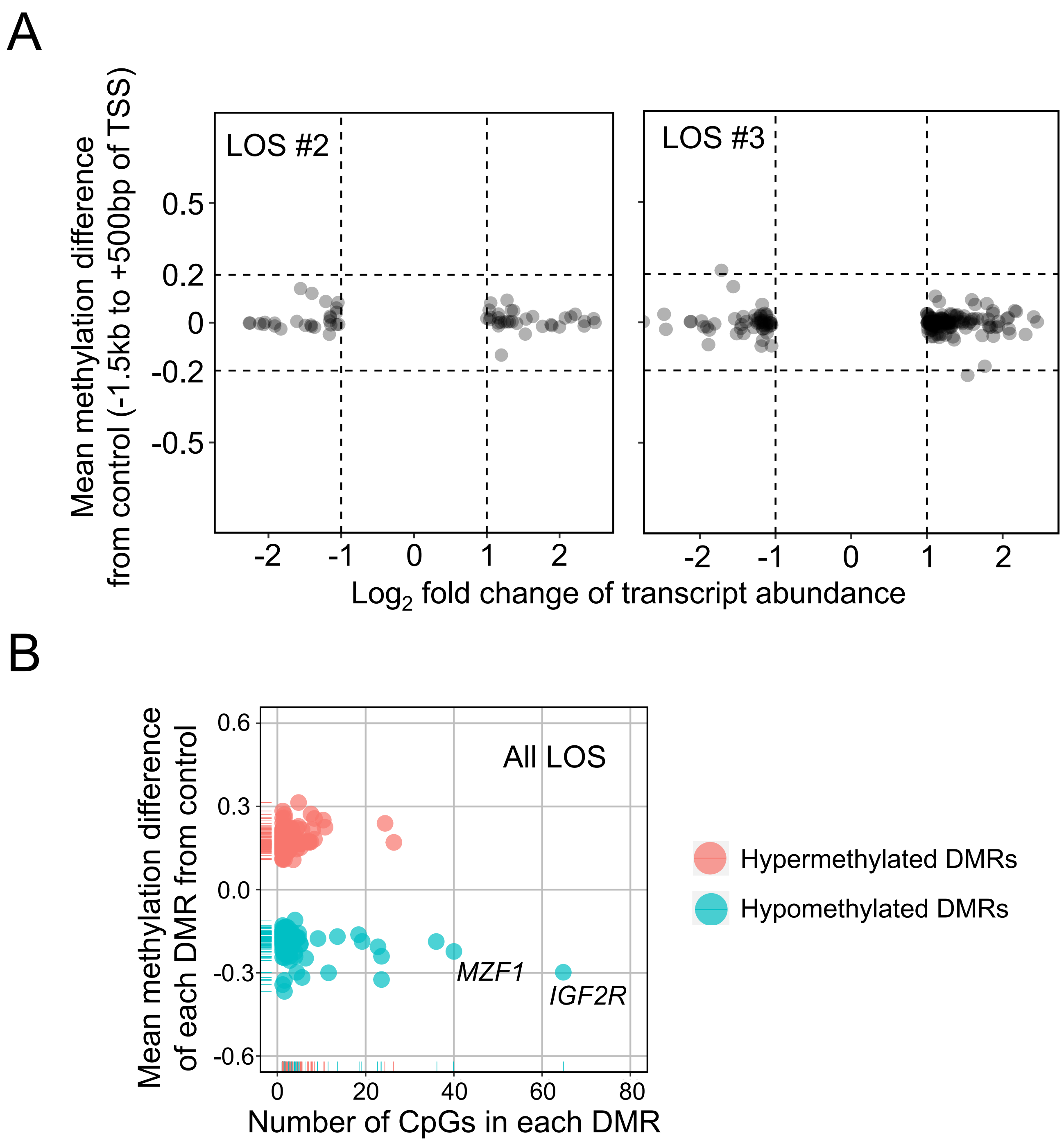

Figure S9. A). Mean methylation level differences of the DEGs between controls and LOS #2 and #3 at the TSS region (-1.5kb to +500bp). Only DEGs that have at least 2-fold change are shown here. **B).** Number of CpGs and the mean methylation difference of each DMR identified when comparing all LOS fetuses to the controls.

Table S1. Summary of the reads alignment and bisulfite conversion rate for each WGBS library

Libraries	Bodyweight	Gestational day	Raw reads	Aligned reads	Aligned reads (%)	Uniquely aligned reads	Uniquely aligned reads (%)	Bisulfite conversion rate (%)
Control #1	392g	Day 104	396,328,924	333,030,504	84.0	267,417,844	67.5	99.73
Control #2	406g	Day 105	428,296,878	331,841,555	77.5	266,934,993	62.3	99.77
Control #3	408g	Day 106	418,901,079	336,561,860	80.3	268,410,454	64.1	99.75
Control #4	416g	Day 106	355,810,944	289,745,138	81.4	229,015,740	64.4	99.56
LOS #1	514g	Day 106	424,025,153	338,241,972	79.8	268,667,106	63.4	99.77
LOS #2	518g	Day 105	431,258,953	333,939,082	77.4	260,337,387	60.4	99.75
LOS #3	620g	Day 106	397,474,455	320,530,155	80.6	254,012,990	63.9	96.48
LOS #4	714g	Day 105	421,457,025	338,500,780	80.3	269,718,690	64.0	99.74

Table S2. Reads coverage at CpG sites

Sample	≥1 read (%*)	≥3 reads (%*)	≥5 reads (%*)	≥8 reads (%*)	≥10 reads (%*)
Control #1	25,422,282 (92.3)	24,523,876 (89.0)	22,835,084 (82.9)	18,144,557 (65.9)	13,953,964 (50.7)
Control #2	25,380,860 (92.2)	24,233,110 (88.0)	22,007,331 (79.9)	16,221,532 (58.9)	11,560,309 (42.0)
Control #3	25,420,791 (92.3)	24,414,185 (88.6)	22,484,754 (81.6)	17,326,183 (62.9)	12,984,193 (47.1)
Control #4	25,260,324 (91.7)	23,354,088 (84.8)	19,582,672 (71.1)	11,582,224 (42.0)	6,717,916 (24.4)
LOS #1	25,431,432 (92.3)	24,491,532 (88.9)	22,732,952 (82.5)	17,931,727 (65.1)	13,732,790 (49.9)
LOS #2	25,427,128 (92.3)	24,505,589 (89.0)	22,772,178 (82.7)	18,057,629 (65.6)	13,958,856 (50.7)
LOS #3	25,426,184 (92.3)	24,550,790 (89.1)	22,958,659 (83.4)	18,522,677 (67.3)	14,501,482 (52.7)
LOS #4	25,416,233 (92.3)	24,368,611 (88.5)	22,334,119 (81.1)	16,895,545 (61.3)	12,340,998 (44.8)

*Percentage of CpGs in the reference assembly UMD3.1 covered by at least 1, 3, 5, 8, and 10 reads.

Table S3. ASM regions identified in bovine fetal skeletal muscle using WGBS

# of DMRs	Chromosome	Start position	End position	Methylation bias toward:	Mannual annotation	Close to known imprinted loci (<1MB)?	Described in cattle before?
1	1	42,531,131	42,531,182	Indicus	1st exon of *CPOX*	No	No
2	1	155,832,527	155,832,651	Taurus	last exon of *PLCL2*	No	No
3	2	108,192,408	108,192,519	Taurus	last exon of *CHPF*	No	No
4	2	133,907,427	133,907,504	Taurus	*CAPZB* intron	No	No
5	2	134,688,529	134,688,625	Indicus	Intergenic	No	No
6	3	14,945,751	14,945,783	Indicus	1st intron of *SYT11*	No	No
7	3	20,813,565	20,813,646	Taurus	upstream of *LOC516742*	No	No
8	3	77,572,982	77,573,138	Taurus	2nd exon of *DIRAS3*	Yes (*DIRAS3* domain)	No
9	3	92,054,107	92,054,197	Taurus	1st exonf of *C3H1ORF177*	No	No
10	3	92,081,371	92,082,016	Indicus	last exon of *TTC22*	No	No
11	3	116,009,370	116,009,556	Indicus	*AGAP1* intron	No	No
12	4	95,065,424	95,065,580	Taurus	*MEST*	Yes (*MEST* domain)	Yes
13	4	95,422,095	95,422,142	Indicus	Intergenic	No	No
14	4	114,468,595	114,468,839	Taurus	*AGAP3* intron	No	No
15	4	119,913,118	119,913,256	Indicus	*PTPRN2* intron	No	No
16	5	118,121,533	118,121,692	Indicus	*TBC1D22A* intron	No	No
17	5	120,976,901	120,976,977	Taurus	~3.5kb downstream of *PIM3*	No	No
18	6	37,508,900	37,510,231	Taurus	*NAP1L5*	Yes (*NAP1L5* domain)	No
19	7	2,485,761	2,486,047	Taurus	*HIST3H2A*	No	No
20	7	44,766,662	44,767,403	Indicus	1st exon of *LOC101903623*	No	No
21	7	46,025,028	46,025,151	Taurus	*SOWAHA*	No	No
22	9	82,472,274	82,473,917	Taurus	1st exon of *PLAGL1*	Yes (*PLAGL1* domain)	Yes
23	9	97,663,961	97,664,174	Taurus	2nd intron of *IGF2R*	Yes (*IGF2R* domain)	Yes
24	10	28,299,731	28,300,013	Indicus	Intergenic	No	No
25	10	74,409,535	74,409,990	Taurus	1st intron of *SYT16*	No	No
26	10	102,886,935	102,887,023	Taurus	last exon of *KCNK13*	No	No
27	10	102,904,503	102,904,679	Indicus	Intergenic	No	No
28	10	102,947,738	102,948,378	Taurus	*NRDE2*	No	No
29	11	1,747,119	1,747,211	Indicus	1st exon of *MALL*	No	No
30	11	70,646,087	70,646,224	Indicus	last exon of *ALK*	No	No
31	11	78,385,525	78,386,123	Taurus	Intergenic	No	No
32	11	87,218,224	87,218,388	Taurus	*HPCAL1* intron	No	No
33	12	11,365,703	11,366,274	Taurus	1st exon of *KBTBD6*	No	No
34	12	16,102,307	16,102,589	Indicus	*ZC3H13* exon	No	No
35	13	39,608,336	39,608,402	Taurus	*SLC24A3 exon*	No	No
36	13	43,033,891	43,034,188	Indicus	*ACSS1*	No	No
37	13	58,033,921	58,039,200	Taurus	*GNASXL* DMR	Yes (*GNASXL*/*GNAS* domain)	Yes
38	13	58,045,896	58,049,131	Indicus	*GNAS* DMR	Yes (*GNASXL*/*GNAS* domain)	Yes
39	13	58,056,771	58,057,077	Taurus	~8kb upstream of *GNAS*	Yes (*GNASXL*/*GNAS* domain)	No
40	13	64,131,680	64,131,870	Taurus	Intergenic	No	No
41	13	65,489,399	65,490,377	Taurus	Intergenic	No	No
42	13	75,947,946	75,948,064	Taurus	Intergenic	No	No
43	15	38,159,369	38,159,492	Taurus	~1.5kb upstream of *CALCA*	No	No

44	15	38,322,072	38,323,223	Taurus	Intergenic	No	No
45	15	38,342,218	38,342,321	Taurus	~2kb upstream of *CALCB*	No	No
46	15	38,411,342	38,412,590	Taurus	overlapping 1st exon of *LOC784945*	No	No
47	17	73,848,859	73,849,139	Indicus	downstream of *RTDR1* and *LOC531152*	No	No
48	18	2,490,132	2,490,721	Taurus	*LDHD*	No	No
49	18	44,226,616	44,226,818	Taurus	Intergenic	No	No
50	18	54,853,816	54,853,887	Indicus	last exon of *SLC8A2*	No	No
51	18	57,589,967	57,590,290	Taurus	*SIGLEC12*	No	No
52	18	58,130,552	58,130,966	Taurus	1st exon of *ZNF613*	No	No
53	18	64,223,889	64,224,080	Taurus	gene body of *MGC157368*	YES (*PEG3* domain)	No
54	18	64,916,693	64,917,122	Indicus	1st exon of *LOC101906235*	No	No
55	19	15,535,373	15,535,514	Taurus	Intergenic	No	No
56	19	23,300,236	23,300,388	Taurus	1st intron of *SLC24A2*	No	No
57	19	23,317,855	23,318,068	Taurus	*SCARF1* exon	No	No
58	19	28,879,630	28,880,069	Taurus	*CCDC42* intron	No	No
59	19	61,552,878	61,553,203	Taurus	Intergenic	No	No
60	19	64,006,701	64,006,992	Indicus	Intergenic	No	No
61	20	2,210,004	2,210,070	Taurus	1st intron of *KCNIP*	No	No
62	20	67,424,767	67,425,257	Taurus	Intergenic	No	No
63	20	70,536,577	70,536,666	Taurus	Intergenic	No	No
64	21	781,530	781,640	Taurus	*MAGEL2*	Yes (*SNRPN* domain)	No
65	21	67,079,426	67,079,951	Indicus	gene body of *BEGAIN*	Yes (*GTL2/DLK1* domain)	No
66	21	67,373,112	67,373,251	Indicus	~3.5kb upstream of *GTL2*	Yes (*GTL2/DLK1* domain)	No
67	21	67,274,019	67,274,308	Taurus	~0.5kb upstream of *DLK1*	Yes (*GTL2/DLK1* domain)	No
68	21	67,448,930	67,449,005	Taurus	*MEG8* intron	Yes (*GTL2/DLK1* domain)	No
69	21	67,717,546	67,717,714	Taurus	Intergenic	No	No
70	21	70,921,482	70,921,682	Taurus	*KIAA0284*	No	No
71	22	681,862	682,055	Indicus	~3.5kb downstream of *VOPP1*	No	No
72	22	53,236,543	53,236,607	Indicus	1st exon of *PRSS42*	No	No
73	22	58,919,867	58,920,123	Indicus	Intergenic	No	No
74	22	61,191,097	61,191,283	Indicus	*SLC41A3* intron	No	No
75	23	9,681,409	9,681,520	Taurus	1st intron of *ARMC12*	No	No
76	23	52,142,619	52,142,708	Taurus	1st intron of *DUSP22*	No	No
77	24	21,945,216	21,945,786	Taurus	Intergenic	No	No
78	25	39,692,219	39,692,665	Taurus	1st intron of *RADIL*	No	No
79	25	39,969,167	39,969,440	Indicus	1st exon of *LOC101907846*	No	No
80	25	42,378,294	42,378,354	Taurus	last exon of *SUN1*	No	No
81	26	30,643,478	30,643,604	Taurus	Intergenic	No	No
82	29	42,176,000	42,176,138	Indicus	Intergenic	No	No
83	29	49,552,793	49,554,961	Taurus	*KCNQ1* intron	Yes (*KCNQ1* domain)	Yes
84	29	50,147,554	50,148,349	Indicus	~2.5kb upstream of *H19*	Yes (*H19/IGF2* domain)	Yes
85	29	50,149,689	50,149,709	Indicus	~1kb upstream of *H19*	Yes (*H19/IGF2* domain)	No
86	29	50,151,516	50,151,573	Indicus	1st exon of *H19*	Yes (*H19/IGF2* domain)	No

Table S4. Methylation status of known imprinted DMRs in human and/or mouse in bovine fetal skeletal muscle

Known imprinted DMRs	ASM in mouse?	ASM in human?	ASM in cow?	ASM in cow supported by:		Methylation bias	DMR coordinates (based on allelic reads)	Notes
				ASM with ≥ 4 WGBS reads/allele	ASM with ≥ 3 WGBS reads/allele			
*AIRN/IGF2R**	✓	✓	✓	✓	✓	Taurus (M)	Chr9: 97,663,961-97,664,174	
CDKN1C promoter	✓	✗	✗					Hypomethylation around TSS
*DIRAS3^*	✗	✓	✓	✓	✓	Taurus (M)	Chr3 :77,572,982-77,573,138	
DLK1	✓	✓	✓	✓	✓	Taurus (M)	Chr21: 67,274,019-67,274,308	
*DLK1-GTL2**	✓	✓	✓	✓	✓	Indicus (P)	Chr21: 67,373,112-67,373,251	
*FAM50B^*	✗	✓	No ortholog					Not annotated in UMD3.1, Blast analyses indicate no ortholog in cattle
*GNAS1A**	✓	✓	✓	✓	✓	Taurus (M)	Chr13: 58,056,771-58,057,077	
*GPR1/ZDBF2**	✓	✓	✗					No evidence of ASM across the intergenic region between *GPR1* and *ZDBF2*
*GRB10**	✓	✓	?					Poor SNPs coverage
H19 promoter	✓	✓	✓	✓	✓	Indicus (P)	Chr29: 50,151,516-50,151,573	
*H19 ICR**	✓	✓	✓	✓	✓	Indicus (P)	Chr29: 50,147,554-50,148,349	
*HERC3/NAP1L5**	✓	✓	✓	✓	✓	Taurus (M)	Chr6: 37,508,900-37,510,231	
*IMPACT**	✓	✗	✗			NA		Hypomethylation around TSS
*INPP5F**	✓	✓	✓	✗	✓	Taurus (M)	Chr26: 40,282,446-40,282,480	
KvDMR1*	✓	✓	✓	✓	✓	Taurus (M)	Chr29: 49,552,793-49,554,961	
*L3MBTL^*	✗	✓	✗			NA		Hypomethylation around TSS
MAGEL2	✓	✓	✓	✓	✓	Taurus (M)	Chr21: 781,530-781,640	
*MCTS2/H13**	✓	✓	?					Poor SNPs coverage
*MEST (PEG1)**	✓	✓	✓	✓	✓	Taurus (M)	Chr4: 95,065,424-95,065,580	
MKRN3	✓	✓	✗					Both parental alleles are partially methylated around TSS
NESP55 (GNAS)	✓	✓	✓	✓	✓	Indicus (P)	Chr13: 58,045,896-58,049,131	
*NESPAS/GNASXL**	✓	✓	✓	✓	✓	Taurus (M)	Chr13: 58,056,771-58,057,077	
NDN	✓	✓	✗					Both parental alleles are hypomethylated around TSS
*NNAT/BLCAP**	✓	✓	?					Poor SNPs coverage
*PEG3/USP29**	✓	✓	?					Poor SNPs coverage
*PEG10/SGCE**	✓	✓	✓	✗	✓	Taurus (M)	Chr13: 11,910,836-11,912,554	
PEG12	✓	No ortholog	No ortholog					Not annotated in UMD3.1, Blast analyses indicate no ortholog in cattle
*PEG13/TRAPPC9**	✓	✓	?					Poor SNPs coverage
*PLAGL1**	✓	✓	✓	✓	✓	Taurus (M)	Chr9: 82,472,274-82,473,917	
*RASGRF1**	✓	✗	✗					Both parental alleles are hypomethylated around TSS
*RB1^*	✗	✓	✗					Both parental alleles are hypomethylated around TSS
*SNURF/SNRPN**	✓	✓	✓	✗	✓	Taurus (M)	Chr21: 25,687-26,167	
*SLC38A4**	✓	?	✗					Hypomethylation around TSS
*ZNF597/NAA60^*	✗	✓	✗					Hypomethylation around TSS
*ZRSR1/COMMD1**	✓	✗	✗					Hypomethylation around TSS

Note:
* Germline DMRs in mouse
^ ASM in human but not in mouse

Supplemental materials and methods

WGBS library preparation and sequencing

Genomic DNA isolated from skeletal muscle of four control and four LOS ~d105 *B. t. taurus* × *B. t. taurus* F_1 fetuses (1) were subject to WGBS analyses. For library preparation, 1μg bovine DNA was subject to sodium bisulfite mutagenesis using NEB EpiMark Bisulfite Conversion Kit (NEB E3318) according to the manufacturer's instructions. Prior to the sodium bisulfite reaction, 5ng (0.5%) unmethylated cl857 *Sam7* Lambda DNA (48,502bp, Promega D1521) was combined with bovine DNA to act as an internal control to monitor the bisulfite conversion rate. The bisulfite conversion rate was calculated as the percentage of thymines sequenced at the cytosine reference positions in the Lambda genome. The WGBS libraries were generated using the NEBNext Ultra DNA library Prep Kit for Illumina (NEB E7370) as per the manufacturer's instructions. Each WGBS library was sequenced in two lanes with 100bp paired-end reads on an Illumina HiSeq 2000 platform. For Control #4 fetus, one lane of the WGBS library was sequenced on an Illumina HiSeq 2500 platform.

WGBS reads trimming and alignment

Raw WGBS read pairs were subject to quality trimming prior to alignment to the reference genome. The Cutadapt program (version: 1.9.1) (2) was used to remove the adaptor sequences in the read pairs. For base quality trimming, DynamicTrim (version 3.1) (3) was used to obtain the longest segment of the read in which each base has <1% error rate. Following quality trimming, WGBS read pairs were aligned to the bovine reference genome assembly UMD3.1 and the Lambda genome using Bismark (version: 0.15.0) (4) with the default parameters. Only uniquely aligned read pairs that had the expected read mate orientation and expected ranges of insert size were retained for further analyses.

Identification of SNPs using WGBS data

In order to distinguish the C>T SNPs in the F_1 fetuses from the C>T substitutions that are caused by sodium bisulfite conversion, Bis-SNP (version: 0.82.2)(5) was used to perform the SNP calling using the WGBS data. In brief, the uniquely aligned WGBS read pairs were subject to several post-alignment processes prior to the SNP calling including PCR duplicates removal, insertions/deletions re-alignment, and base quality recalibration. Known insertions/deletions (indel) and known SNPs downloaded from Ensembl and RefSeq FTP sites were supplied for the indel realignment and the base quality recalibration (ftp://ftp.ensembl.org/pub/release-78/variation/vcf/bos_taurus/; ftp://ftp.ncbi.nlm.nih.gov/snp/organisms/cow_9913/VCF/; ftp://ftp.ncbi.nlm.nih.gov/snp/organisms/cow_30522/). In addition, the SNPs identified from the DNA sequencing data (6) of the sire of the *B. t. indicus X B. t. taurus* F_1 fetuses were also supplied for the base quality recalibration process. After processing the uniquely aligned WGBS read pairs, Bis-SNP was used to identify SNPs with the exclusion of first 3bp and last 2bp of the both read mates using the option "*-trim5 3 –trim3 2*" as these read positions were associated with strong methylation bias (Fig. S1).

Determination of the methylation level of each CpG site

Only CpG site that show consensus CpG context for both parental alleles (*i.e.,* do not overlap any SNPs identified by Bis-SNP) were used for the subsequent analyses. As CpG methylation is symmetric, the methylation level was determined using the reads from both forward and reverse strands covering the same symmetric CpG site. The methylation level was calculated as: (number of "C" reads)/(number of "C" reads + number of "T" reads). For most analyses, only CpGs covered by at least by five reads were used for the calculation of the methylation level. The exception is the allele-specific methylation analyses, in which only CpGs covered at least by 8 reads (four reads per parental allele) were included for the statistical test. For reads with PCR duplicates, only one of them was randomly choosed for the determination of the methylation level.

Identification of the ASM regions

Heterozygous SNPs of the fetuses were obtained from two sources for the determination of parental allele origin: 1) SNPs identified from the WGBS data using Bis-SNP (5); 2) SNPs identified from the RNA-seq

data of the F_1 hybrids (6). The WGBS read pairs overlapping SNPs were assigned to their parental origins based on the genotype of the *B. t. indicus* sire (6). If the SNP included a cytosine, its bisulfite converted form thymine was also considered. Further, the allelic WGBS reads were pooled from four controls to determine the sequencing depth and only CpGs that had at least 4× coverage of each allele were used for Fisher's exact test. To estimate the FDR, WGBS read pairs overlapping SNPs were permutated for their parental origin by randomly assigning them to either *B. t. indicus* or *B. t. taurus* alleles. The ASM for each CpG in the permutated dataset was assessed using Fisher's exact test. The permutation was performed five times and the numbers of identified ASM CpGs at different p-value cutoff were averaged. ASM CpGs were identified using a p-value of 0.01, resulting 109,794 ASM sites with a FDR of ~5% as simply by chance 5507 ASM sites can be identified in the permutated datasets. Lastly, the identified ASM sites were clustered into regions as previously described (7). In brief, significant ASM sites within 2kb were merged as DMR candidates. For each candidate, at least five ASM sites were required and at least 75% CpGs should show consistent methylation bias toward the same allele. The clustered DMRs were also manually inspected and annotated.

Association between DEGs and DMRs in LOS fetuses

Methylkit R package (8) was used to perform the hierarchical clustering of the methylation profiles using 1-Pearson's correlation distance. The DMRs between each LOS fetus and all four controls was identified using Bsseq R package (9) with the default parameters except that only CpGs with at least 5 reads coverage for each fetus were included for analyses. For comparison, Bsseq was also used to identify DMRs between all four LOS fetuses and the controls.

RNA-seq data of the control and LOS fetuses were obtained from one of our previous studies and were processed as previously described (6, 10). DEGs between each LOS fetus and all four controls were determined using the edgeR package (11, 12). In brief, for each LOS fetus, a DGElist was created with the gene read counts of the four controls and the LOS fetus. Following normalization (*i.e.,* function "calcNormFactors") and parameters estimation (functions "estimateGLMTrendedDisp" and "estimateGLMTagwiseDisp"), functions "glmFit" and "glmLRT" were used to identify genes with differential transcript abundance between the LOS and the mean of the four controls (FDR < 0.05). To determine whether any DMRs are associated with DEGs, 5kb or 20kb regions surrounding the DEGs (including the gene body) were used to intersect with the DMRs using "intersectbed" from bedtools (13). The methylation level of the TSS region (-1.5kb to +500bp) was calculated by averaging the methylation level of all CpGs with at least five reads coverage.

WGCNA and KEGG analyses

A weighted gene co-expression network was constructed for the skeletal muscle of the four control and four LOS fetuses using "blockwiseModule" function from the WGCNA package (14). Further, the identified network modules were linked to the external trait (*i.e.,* bodyweight) to determine which module is significantly correlated to the bodyweight. In addition, KEGG pathway analyses for the significant network modules were performed using the Database for Annotation, Visualization and Integrated Discovery (DAVID) (15).

Genomic annotations

The TSS and TES of the annotated genes were obtained from Bovine Genome Database (http://bovinegenome.org)(16) and were curated with the RNA-seq data of brain, kidney, liver, and skeletal muscle using cuffmerge (17). Annotation of CpG islands was obtained from UCSC genome browser (18), in which the CpG islands are defined as genomic regions that fulfill the following criteria: 1) > 200bp, 2) GC content ≥ 50% and 3) the ratio of the observed CpGs to the expected CpGs (O/E) > 0.6.

Promoters are defined as -2kb to +500bp of the TSS (19). High CpG Promoters (HCP) promoters contain a 500bp interval with: 1) GC content ≥ 55% and 2) the O/E ratio ≥ 0.6 (20). Low CpG Promoters (LCP) promoters do not contain a 500bp interval with the O/E ratio ≥ 0.4 (20). The rest of the promoters are classified as Intermediate CpG Promoters (ICP) promoters (20). The formula used for calculation of the O/E ratio of a sequence segment is as follows: number of CpGs * sequence length / (number of C * number of G) (21).

Allelic expression analyses of *AIRN*

Both DNA and cDNA of the F_1 fetuses and DNA of the sire were amplified by PCR followed by Sanger sequencing to ascribe the parental origin of the transcript. Nucleic acid isolation, cDNA synthesis, and PCR amplifications were as previously reported (10). The primers used are as follows: forward (5'-AGAAACGGGCACATTCA-3') and reverse (5'-GGCAATATTAGAACAGCTAAGAG-3').

Statistical analyses and data visualization

Statistical analyses such as Fisher's exact test were implemented with R (http://www.r-project.org/). Fig. 2A and C and Fig. S2C-D were generated with R function "heatmap.2". Fig. 3C and 5B were generated using the "computeMatrix" and "plotProfile" functions in deepTools (22). For Fig. 4B-D, Fig. 5C, Fig. S5, Fig. S6A, C, and E, bed files were converted to the bigwig format using the "bedGraphToBigWig" from UCSC Genome Browser database and visualized as custom tracks in the Integrative Genomics Viewer (IGV) (23).

Supplemental References

1. Seisenberger S, *et al.* (2012) The dynamics of genome-wide DNA methylation reprogramming in mouse primordial germ cells. *Molecular cell* 48(6):849-862.
2. Martin M (2011) Cutadapt removes adapter sequences from high-throughput sequencing reads. *EMBnet.journal* 17(1):10-12.
3. Cox MP, Peterson DA, & Biggs PJ (2010) SolexaQA: At-a-glance quality assessment of Illumina second-generation sequencing data. *BMC bioinformatics* 11:485.
4. Krueger F & Andrews SR (2011) Bismark: a flexible aligner and methylation caller for Bisulfite-Seq applications. *Bioinformatics* 27(11):1571-1572.
5. Liu Y, Siegmund KD, Laird PW, & Berman BP (2012) Bis-SNP: Combined DNA methylation and SNP calling for Bisulfite-seq data. *Genome biology* 13(7):R61.
6. Chen Z, *et al.* (2016) Global assessment of imprinted gene expression in the bovine conceptus by next generation sequencing. *Epigenetics : official journal of the DNA Methylation Society* 11(7):501-516.
7. Xie W, *et al.* (2012) Base-resolution analyses of sequence and parent-of-origin dependent DNA methylation in the mouse genome. *Cell* 148(4):816-831.
8. Akalin A, *et al.* (2012) methylKit: a comprehensive R package for the analysis of genome-wide DNA methylation profiles. *Genome biology* 13(10):R87.
9. Hansen KD, Langmead B, & Irizarry RA (2012) BSmooth: from whole genome bisulfite sequencing reads to differentially methylated regions. *Genome biology* 13(10):R83.
10. Chen Z, *et al.* (2015) Characterization of global loss of imprinting in fetal overgrowth syndrome induced by assisted reproduction. *Proceedings of the National Academy of Sciences of the United States of America* 112(15):4618-4623.
11. Robinson MD, McCarthy DJ, & Smyth GK (2010) edgeR: a Bioconductor package for differential expression analysis of digital gene expression data. *Bioinformatics* 26(1):139-140.
12. McCarthy DJ, Chen Y, & Smyth GK (2012) Differential expression analysis of multifactor RNA-Seq experiments with respect to biological variation. *Nucleic acids research* 40(10):4288-4297.
13. Quinlan AR & Hall IM (2010) BEDTools: a flexible suite of utilities for comparing genomic features. *Bioinformatics* 26(6):841-842.
14. Langfelder P & Horvath S (2008) WGCNA: an R package for weighted correlation network analysis. *BMC bioinformatics* 9:559.
15. Huang DW, *et al.* (2007) DAVID Bioinformatics Resources: expanded annotation database and novel algorithms to better extract biology from large gene lists. *Nucleic acids research* 35(Web Server issue):W169-175.
16. Elsik CG, *et al.* (2015) Bovine Genome Database: new tools for gleaning function from the Bos taurus genome. *Nucleic Acids Res*.
17. Trapnell C, *et al.* (2010) Transcript assembly and quantification by RNA-Seq reveals unannotated transcripts and isoform switching during cell differentiation. *Nat Biotechnol* 28(5):511-515.
18. Rosenbloom KR, *et al.* (2015) The UCSC Genome Browser database: 2015 update. *Nucleic Acids Res* 43(Database issue):D670-681.
19. Liao J, *et al.* (2015) Targeted disruption of DNMT1, DNMT3A and DNMT3B in human embryonic stem cells. *Nat Genet* 47(5):469-478.
20. Mikkelsen TS, *et al.* (2007) Genome-wide maps of chromatin state in pluripotent and lineage-committed cells. *Nature* 448(7153):553-560.
21. Gardiner-Garden M & Frommer M (1987) CpG islands in vertebrate genomes. *J Mol Biol* 196(2):261-282.
22. Ramirez F, Dundar F, Diehl S, Gruning BA, & Manke T (2014) deepTools: a flexible platform for exploring deep-sequencing data. *Nucleic acids research* 42(Web Server issue):W187-191.
23. Robinson JT, *et al.* (2011) Integrative genomics viewer. *Nature biotechnology* 29(1):24-26.
